# Fragmentation of nest and foraging habitat affects time budgets of solitary bees, their fitness and pollination services, depending on traits: Results from an individual-based model

**DOI:** 10.1371/journal.pone.0188269

**Published:** 2018-02-14

**Authors:** Jeroen Everaars, Josef Settele, Carsten F. Dormann

**Affiliations:** 1 Department of Computational Landscape Ecology, Helmholtz Centre for Environmental Research - UFZ, Leipzig, Germany; 2 Department of Ecological Modelling, Helmholtz Centre for Environmental Research - UFZ, Leipzig, Germany; 3 Department of Community Ecology, Helmholtz Centre for Environmental Research - UFZ, Halle, Germany; RMIT University, AUSTRALIA

## Abstract

Solitary bees are important but declining wild pollinators. During daily foraging in agricultural landscapes, they encounter a mosaic of patches with nest and foraging habitat and unsuitable matrix. It is insufficiently clear how spatial allocation of nesting and foraging resources and foraging traits of bees affect their daily foraging performance. We investigated potential brood cell construction (as proxy of fitness), number of visited flowers, foraging habitat visitation and foraging distance (pollination proxies) with the model SOLBEE (simulating pollen transport by solitary bees, tested and validated in an earlier study), for landscapes varying in landscape fragmentation and spatial allocation of nesting and foraging resources. Simulated bees varied in body size and nesting preference. We aimed to understand effects of landscape fragmentation and bee traits on bee fitness and the pollination services bees provide, as well as interactions between them, and the general consequences it has to our understanding of the system. This broad scope gives multiple key results. 1) Body size determines fitness more than landscape fragmentation, with large bees building fewer brood cells. High pollen requirements for large bees and the related high time budgets for visiting many flowers may not compensate for faster flight speeds and short handling times on flowers, giving them overall a disadvantage compared to small bees. 2) Nest preference does affect distribution of bees over the landscape, with cavity-nesting bees being restricted to nesting along field edges, which inevitably leads to performance reductions. Fragmentation mitigates this for cavity-nesting bees through increased edge habitat. 3) Landscape fragmentation alone had a relatively small effect on all responses. Instead, the local ratio of nest to foraging habitat affected bee fitness positively through reduced local competition. The spatial coverage of pollination increases steeply in response to this ratio for all bee sizes. The nest to foraging habitat ratio, a strong habitat proxy incorporating fragmentation could be a promising and practical measure for comparing landscape suitability for pollinators. 4) The number of flower visits was hardly affected by resource allocation, but predominantly by bee size. 5) In landscapes with the highest visitation coverage, bees flew least far, suggesting that these pollination proxies are subject to a trade-off between either longer pollen transport distances or a better pollination coverage, linked to how nests are distributed over the landscape rather than being affected by bee size.

## 1. Introduction

Wild and solitary bees, important crop pollinators in agriculturally dominated landscapes [1, 2, 3] and essential pollinators of many wild plants [4, 5], are clearly declining worldwide [6–8]. Agricultural intensification limits solitary bees to live on resource islands in an unrewarding matrix [9, 10], because dominant crops provide hardly any foraging resources (e.g. wheat, maize, rice) and separate nest from foraging habitat [11], thereby creating a mosaic of fields and natural elements. Such fragmentation at local scales is expected to affect the distribution and pollination potential of solitary bees, which are central-place foragers and therefore prefer foraging resources within several hundred meters from their nest. Natural supply of pollination services by wild pollinators is important for production of many non-dominant crops [12, 13] and protecting natural habitats near such crop fields seems to be a key solution to secure it [14, 15–17]. However, defining how landscape mosaics in agriculturally dominated landscapes can be optimized for wild bees remains a complex subject [16]. We need to understand how wild bees interact with the landscape to improve landscape configuration and meet all needs of vital wild bee populations.

It is clear that wild bee abundance and species diversity at the landscape scale are positively affected by foraging habitat availability [18–20] and nest habitat availability [20–22]. Daily area requirements of solitary bees depend on the distance between nesting and foraging resources [11, 17] and hence on landscape fragmentation.

Effects of habitat fragmentation, i.e. the process of spatial separation of habitat patches independent of reduced habitat availability [23], is insufficiently studied for bees and not fully understood. Fragmentation affects bees on at least two different scales. First, fragmentation reduces connectivity of nest sites at larger scales (dozens to hundreds of kilometres) and therefore gene-flow between isolated populations [24]. At the scale of the agricultural patch mosaic (several hectares to a few kilometers, hereafter termed landscape fragmentation), fragmentation reduces connectivity between nest and foraging sites, affecting daily foraging success. This is of high research interest due to the inevitable consequences for pollination. At the local habitat patch scale (meters), bees may hardly respond to fragmentation [25]. Habitat fragmentation studies have considered the size of the fragments rather than fragmentation itself [26, 27, 28]. Meadow isolation reduced the number of brood cells of cavity-nesting bees in trap nests [29]. In spite of a general effect of habitat fragmentation on bees, effects may be species-specific and trait-dependant [4, 9, 27]. The performance of bees in agricultural field mosaics is still difficult to forecast, despite current knowledge on the effect of foraging traits.

Two traits are especially relevant for how solitary bee species interact with the landscape and respond to habitat fragmentation: nesting preference and body size [30, 31]. Nesting preference determines the home location of their central-place foraging activities, in turn affecting their spatial distribution in the landscape. Body size affects foraging traits such as velocity or capacity for carrying pollen [32]. The few studies that investigated how wild bee nesting preference and body-size affect responses to fragmentation show contrasting results. Bees nesting above ground are more sensitive to disturbance factors and they are more affected by patch isolation [30]. At the same time, they can also be more abundant in small patches [31]. Average body size of wild bee communities was reported to be larger in more isolated [33] and smaller patches [31], while also the opposite, relatively more small bees in isolated or small patches has been found [28, 34]. Life-history traits, including nesting preference and body size, are often correlated in data sets, obscuring clear effects of single traits [30].

Our mechanistic understanding of effects of single traits on the way wild bees respond to fragmentation is still incomplete. Small bee species are on the one hand expected to be mostly negatively affected in highly fragmented landscapes, since they may not be able to cross the matrix without foraging resources in agriculturally dominated landscapes [21]. Bees of intermediate size are expected to be affected when they are mobile enough to leave a large patch, but not mobile enough to reach a distant foraging patch [35]. On the other hand, all bees may easily survive in a network of patches that are available within their foraging range [19, 36], and especially small bees with lower resource requirements may profit. Generally however, bees are good flyers (large more than small) and probably most of them are able to cross an agricultural matrix of hundreds of meters and able to reach distant resource patches.

To investigate the performance of different solitary bee types in fragmented landscapes we use the ecologically-detailed model SOLBEE [37]. SOLBEE is an individual-based, spatially-explicit model to simulate solitary bees foraging for pollen in the landscape. Individual-based or agent-based models (IBM/ABM) are a well-established approach to investigate competition and resource depletion in space and time, such as solitary bees that adapt their movement to local changes made by other solitary bees. SOLBEE incorporates behaviour and decision-making known for solitary bees and allows investigation of species traits (e.g. body size and nesting preference). The model is a resource competition model during one day in a 100 ha landscape, which measures performance parameters at the bee level as proxies for fitness and pollination.

In this study we investigate small-scale fragmentation in landscape mosaics (scale of one kilometre), where foraging resources and fragmented edge habitat affect daily foraging performance and pollination services. Bees differing in body size and nesting preference are expected to differ in their performance. Our goal is to gain a better understanding of how different bees cope with local spatial resource distribution and how spatial pollination is affected. Specifically we ask: What is the effect of landscape fragmentation on daily performance, and can we observe both the negative and positive effects as shown by field studies? How does local landscape fragmentation affect pollination services? Do different bee types (differing in body size and nesting preference) respond differently to landscape fragmentation? Do different bee types provide pollination services differently? Do pollination proxies provide a consistent pattern or are there trade-offs? Can we define optimal conditions in fragmented landscapes for 1) bee fitness (daily performance of solitary bees) and 2) pollination, which 3) can be generalized for different bee types?

## 2. Methods

### 2.1. Study system

Solitary bees are the largest sub-group of wild bees with about 14,000 species worldwide [38]. They span a much wider range of body sizes than honeybees and bumblebees and are therefore considered as a better group of model species for studying foraging performance of pollinators in fragmented landscapes. Solitary bees differ considerably from eusocial bees in foraging behaviour and efficiency, such as in length of flower visits and number of flower visits per time unit [28, 39, 40]. They forage alone and therefore face limited knowledge of resource locations and their quality, while communication between eusocial bees can lead to near-optimal foraging [41, 42]. Solitary bees are also characterized by their shifted focus towards collection of pollen for provisioning their offspring, while eusocial bees spend a large part of their time foraging for nectar [43].

The traits ‘body length’ and ‘nesting preference’ were chosen to represent a large range of solitary bee species, without making the model too complex (e.g. with plant family preference). Body lengths of wild bees normally vary in most parts of the world between 4 mm and 28 mm [43]. Nesting preference of solitary bees can be coarsely classified into below-ground soil-nesting bees and above-ground cavity-nesting (often wood-nesting) bees [11]. In agriculturally dominated landscapes, nesting substrates for wood-cavity nesters are found at structures with shrubs, trees and dead wood, while open spots with bare ground for soil-nesting bees can be found in unmanaged sites such as grasslands and set-aside fields.

As a simplification, we considered six bee types, which are representative of the variability found over a wide range of species (see [37] for examples). These six types are a combination of three body sizes (6, 12 and 24 mm body length) and two nesting preferences (soil-nesting and wood- or cavity-nesting bees). Bumblebees (most workers being 12–18 mm) do not live solitarily and are not directly represented by our model.

### 2.2. Model description

**Fig 1:**
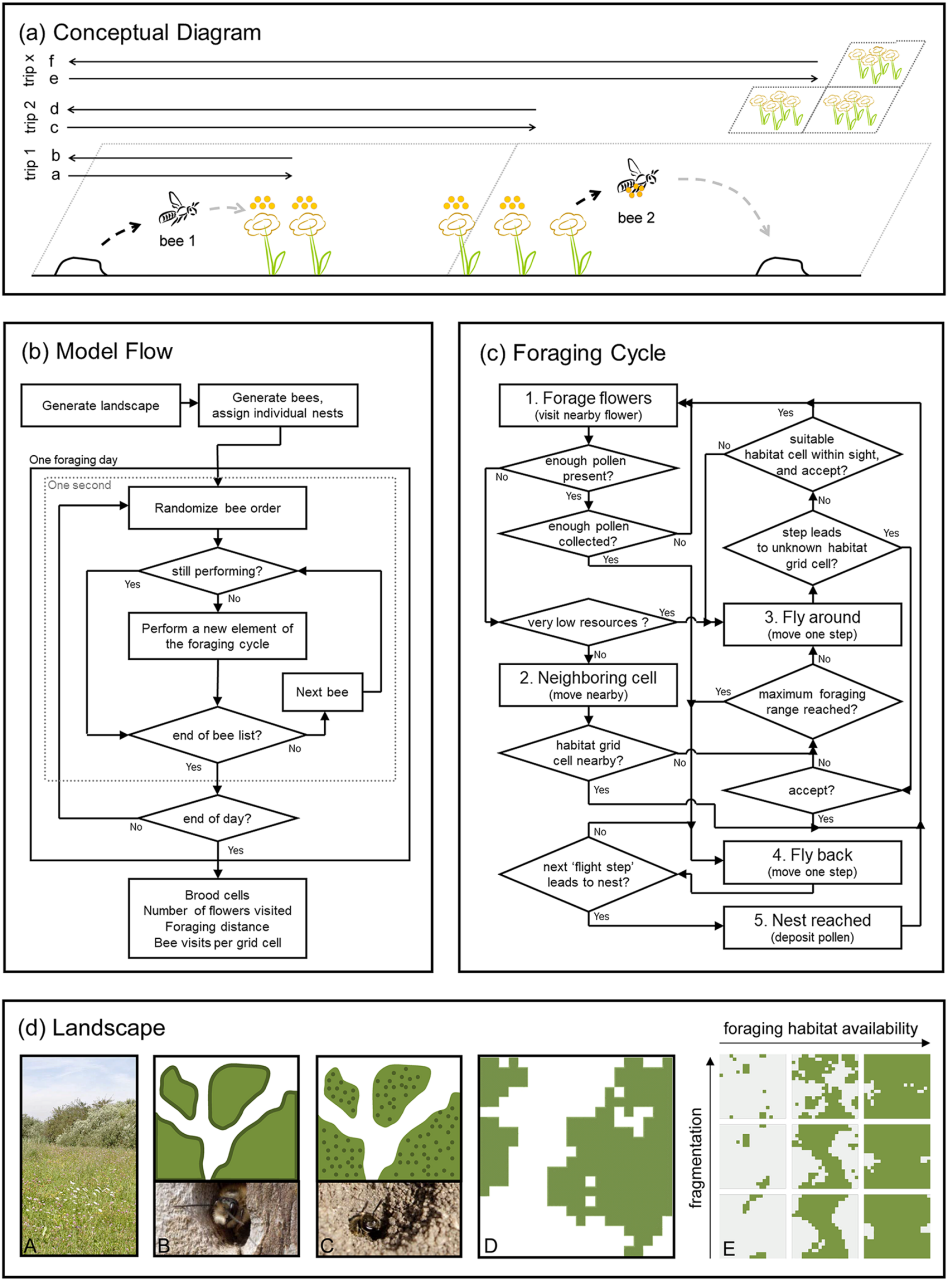
Four panels illustrating model concepts and model flow. First panel (a) shows a conceptual diagram, illustrating how landscape grid cells with flowers are used by two bees that dislocate pollen from flowers to their nests during different foraging trips. On the first foraging trip a solitary bee (bee 1) flies from the nest (black dashed arrow from the small hill on top of the soil) to the most nearby flower (grey dashed arrow) to collect pollen (dots) and returns to the nest when a full pollen load is collected, all occurring in the left grid cell. At the same time, another bee (bee 2) in a neighbouring grid cell transports pollen from other nearby flowers (black dashed arrow) to its nest (grey dashed arrow). On the next foraging trip (trip 2) both bees could be competing for pollen at the same flowers. On a later foraging trip (trip x) both bees may need to look for more distant flowers (the three grid cells with flowers in the background) to collect pollen. The three foraging trips back and forth (a-f) are from the perspective of bee 1 to illustrate how it uses multiple grid cells (lines are dimensionless). Flower and pollen information is in the model aggregated at the grid cell level as simplification (e.g. distance to nearest flower and its remaining pollen is estimated by calculation), while individual bees are followed during all time steps giving an average pollen collection performance for all bees at the end of the simulation. The second panel (b) illustrates the basic flow of the model. The third panel (c) illustrates relationships between the five behavioural modules as flow diagram. “Cell” always stands for a grid cell with foraging habitat and a “step” is a flight unit in a correlated random walk (can be multiple grid cells). Further explanations of the model elements can be found in the main text. The fourth panel (d) illustrates landscape concepts with lighter shades of green being foraging habitat and darker shades of green being nesting habitat (white/grey represents matrix without bee habitat). Solitary bees live in large fields with foraging habitat surrounded by field edges with scrubs (panel A). In these edges with scrubs, wood-nesting bees find their nest habitat (panel B), while soil-nesting bees find their nest habitat everywhere as long as there is some bare soil (panel C). Simulated landscapes (1 by 1 km) assume a minimal patch size of 50 by 50 meters (panel D) and can have different levels of landscape fragmentation which works out differently for landscapes with different foraging habitat availability as nine example landscapes show (panel E). Photographic material by the main author, also available on Wikimedia.

The simulation model SOLBEE mimics solitary bees that forage and compete for pollen in agriculturally dominated landscapes and is capable of reproducing realistic foraging behaviour and measurable output. It comprises allometric rules to link foraging traits to body size, behavioural rules to describe movement and decision making, and rules for pollen depletion of flowers, all parameterized for solitary bees. The model SOLBEE has been thoroughly described, parameterized, verified and roughly validated with independent field observations (see [37], and below for more details) and is suitable for assessing our questions. There is an extensive documentation of the biological support for the parameter values, for important assumptions and for output values [37] as well as a complete ODD (Overview, Design concepts and Details [44, 45]) protocol for the model (S1 Appendix), summarised here and visually supported with Fig 1.

#### 2.2.1. Purpose of the model

This rule- and individual-based model SOLBEE simulates spatial pollen foraging behaviour of central place foragers and is designed to be applicable to multiple solitary bee species foraging individually for pollen (Fig 1a). One simulation measures the mean performance of a group of individual bees sharing the same focal traits, in terms of fitness (potential number of brood cells based on collected pollen) and pollination activity (flowers visited, percentage visited foraging habitat, mean distance flown from the nest). The aims of the model are: 1) understanding how solitary bees interact with landscape structure by comparing their performance in different landscapes, 2) understanding how solitary bees with different traits interact with landscape structure by comparing the performance of groups (differing in focal traits) and 3) to give insight into the solitary bee's needs (concerning resource distribution) within a landscape and the consequences for pollination from the perspective of the bee. To reveal performance differences among groups caused by fragmentation only, the maximum expected number of brood cells per bee (per capita resource availability) is set equally. Bee densities (number of individuals) of each type are therefore scaled with body length and the amount of pollen in the landscape.

#### 2.2.2. Entities, state variables and scales

The model comprises a spatially explicit grid-based landscape with a given resource distribution and a community of pollen-collecting bees. A list of all parameters (*italic* in the main text) and their values is given in Table 1.

**Table 1:** List of model parameters with definitions, units and simulated values.

Parameter	Unit	Definition	Value(s)
***Landscape***:			
landscape stochastic factor		initial number for pseudo-random number generator	20 random replicate numbers
landscape size	m	spatial extent of simulated landscape, length	1000
landscape element size	m	length of most detailed landscape element (grain size of coarse grid)	50
landscape detail	m	grain size of final grid	5
foraging habitat availability		proportion of landscape suitable as foraging habitat	0.05, 0.15, …, 0.85, 0.95
landscape fragmentation		reverse Hurst exponent (terrain smoothness), habitat fragmentation	0.05, 0.15, …, 0.85, 0.95
flower density	m^-2^	number of flowers per square meter	50 (25)
pollen per flower	mm^3^	pollen volume available per flower per day	0.5 (1.5)
pollen availability		proportion of pollen extractable from flower per pollinator visit	0.3 (0.2)
landscape quality		max. brood cell number per individual based on pollen in landscape	30
***Bee***:			
body length	mm	body length of individuals	6, 12, 24
nesting preference		category of nesting preference	wood-nesting, soil-nesting
flower memory		min. number of most recently visited flowers that can be memorized	3
habitat cell memory		number of most recently visited habitat units that can be memorized	10
flight path tortuosity		density parameter (from wrapped Cauchy distribution) determining distribution (hence proportion small) of turning angles during flight	0.9
lower patch leaving threshold		relative quality below which a bee must leave the habitat grid cell	0.5
upper patch leaving threshold		relative quality above which a bee must stay in the habitat grid cell	0.9
time at the nest	s	time spent at the nest for non-foraging activities	30
flytime	s	total time of activity during a foraging day	14400
***Bee (dependent)***:			
pollen per brood cell	mm^3^	pollen volume needed to build one brood cell	9.1, 43.1, 204.7
pollen capacity per bee	mm^3^	max. amount of pollen carried per bee per foraging bout	0.91, 4.31, 20.47
velocity medium/low	m·s^-1^	flight velocity in foraging habitat	0.60, 1.41, 3.03
velocity high	m·s^-1^	flight velocity in matrix habitat	2.79, 4.10, 6.72
handling time per flower	S	time required for removing pollen from flower	29.8, 7.9, 2.1
perception distance	m	distance radius within bees recognize habitat cells with flowers	27.9, 41.0, 67.2
length of flight units	m	mean length of a flight unit of which a flight path is built	14.0, 20.5, 33.6
general return distance	km	the distance for which probability of obligatory return is 0.5	0.061, 0.641, 6.761
far return distance	km	the distance for which probability of obligatory return is 0.9	0.120, 1.387, 16.017
ignorance		probability of (non)ignoring a flower location within sight or at present location, inverse of habitat cell memory	0.1

Model parameters are separated by those for defining landscapes and those for defining bees. Nine bee parameters directly depend on *body length*, yielding three calculated values for each and given here for clarity, as well as one parameter depending on *flower memory*. The values used for simulating adjusted vegetation are given in brackets.

A model landscape requires at least habitat units that can be used for nesting, foraging, both or none (matrix), and for our specific questions with a pre-defined landscape fragmentation. We use the midpoint displacement algorithm [46] to stochastically (*landscape stochastic factor*) generate a coarse grid (detail set by a grain size of 50 m, defined in *landscape element size*) with a given proportion of vegetation units suitable for foraging (*foraging habitat availability*) and a given *landscape fragmentation* that separates vegetation units from non-vegetation units (Fig 1d panel D and E, 20 by 20 grid cells). We rescale the landscape to a finer resolution (grain size of 5m, defined in *landscape detail*) to define small homogeneous habitat units that can be visited by foraging bees and be used as nest habitat. This final grid has a spatial extent of 1 km (*landscape size*) subdivided into 200 by 200 grid cells.

The vegetation for each habitat unit is simplified with a "single generic type of plant", described by *flower density*, amount of *pollen per flower*, and the proportion of pollen that is available per pollinator visit (*pollen availability*, limits instant flower depletion) with parameter values from published data. This generic habitat with flowers represents a natural situation for wild bees (patches with flowering crops, which are extremes in the sense of flower densities and flower sizes, are not further considered here). The vegetated habitat is further split up in edge (width set by *landscape detail*, hence 5 m wide strips) and interior to represent two types of nesting habitat (Fig 1d panel A). Edge and interior are both used for nesting (and foraging) by soil-nesting bees (nest at bare spots in the vegetation, Fig 1d panel C), while wood-nesting bees use field edges only (where in real landscapes shrubs and trees grow, Fig 1d panel B) to nest in. The *landscape quality* for bees (maximum potential number of brood cells per individual per bee, here with a value of 30, representing a clear excess of pollen in the landscape) is used to regulate initial bee densities in the landscape (see also section 2.2.3 and details in section 5 of ODD in S1 Appendix). We track the remaining pollen volume and the number of bee visits per grid cell during simulation. The time horizon (*flytime*) is 14,400 time steps, each representing one second (foraging period of four hours).

Bees are characterized by *body length* and *nesting preference*, i.e. nesting in dead wood and other cavities (hereafter ‘wood nesting’) or in the upper soil layer (hereafter ‘soil nesting’). Nesting preference determines the fixed nest location for each individual bee. Empirical allometric rules (S1 Appendix, Table C) are applied to link foraging traits (*pollen per brood cell*, *general return distance*, *far return distance*, *velocity medium/low*, *velocity high*, *handling time per flower*), and subsequently derived traits (*pollen capacity per bee*, *perception distance*, *length of flight units*) to body size. Other behavioural traits needed for foraging (*flower memory*, *habitat cell memory*, *upper patch leaving threshold*, *lower patch leaving threshold*, *flight path tortuosity*, *ignorance*, *time at the nest*) were also parameterized with literature data [37]. All traits of bees remain constant during simulation (calculated values given in Table 1). Specification of state variables of bees can be found in the supplementary material (S1 Appendix, ODD section 2).

The mean number of brood cells per bee (potential brood success based on collected pollen) represents a proxy for number of offspring and fitness. To calculate this number from collected pollen, we correct for body size (dividing pollen volume by pollen per brood cell, see values in Table 1). As proxies for pollination performance we track the number of flower visits and the mean distance from the nest at return. These three responses at the bee level are given as mean averaged over all bees in the community at the end of the simulation. At the landscape level, we calculate at the end of the simulation the percentage of foraging habitat that was visited by at least one bee (percentage of grid cells with one or more visits) which we also use as indicator for pollination performance. No further population level variables are considered.

#### 2.2.3. Process overview and scheduling

The initialization phase consists of generating a landscape with foraging habitat and defining a bee community (with individuals of one type) and setting individual nest locations (Fig 1b). To allow direct comparison of the performance of the six bee types, we assumed equal resource availability per individual, having for a certain landscape either many small or few large bees. To focus on landscape fragmentation and avoiding to model food loss, we decided to have *foraging habitat availability* also increase the number of individuals. This assumption of bee population being in balance with the available resources, allows to focus on spatial allocation differences (of pollen, and bee nests). The calculation of the number of bees within a landscape includes all these variables and uses pollen volume (mm^3^) as common unit. In short is calculated by dividing the pollen volume present in the landscape by the pollen volume that should be available per individual. The numerator is calculated by multiplying *foraging habitat availability*, landscape area (1 km^2^), *flower density* and *pollen per flower* (i.e. x flowers multiplied by pollen volume) and the denominator by multiplying *landscape quality* and *pollen per brood cell* (volume for x brood cells). The *landscape quality* defines the maximum potential number of brood cells per individual, as being equal for all bee types in all landscapes. The initially calculated number of individuals (per bee community of bees of one type) remains constant during simulation (one foraging day without population dynamics). After initialization, a foraging day (time horizon *flytime*) starts for each individual with five types of behaviour in a foraging cycle (modified after [47]). The behaviour of one individual is strictly sequential in time, and lasts at least for one second (discrete time steps, ‘time penalties’, Table 2). The five behaviours in the foraging cycle (Fig 1c, details in S1 Appendix) are: FORAGE FLOWERS (collect pollen on flowers); NEIGHBORING CELL (fly to a suitable neighbouring landscape grid cell); FLY AROUND (fly around and visually search for unknown foraging areas); FLY BACK (fly back to nest); NEST REACHED (deliver the pollen to the nest). When an individual performs a behaviour, foraging performance state variables are changed in the first second (and for simplification the bee is set to a "still performing" pause mode). Individuals are processed in random order each model second and start a new behavioural element when they are not in "still performing" mode. The sequential approach assumes that always a single individual reaches a flower as first being allowed to remove resources. The randomization ensures that 'being first' is a process of chance. This sequence is repeateduntil a certain foraging time (*flytime*) is completed after which all activities are stopped instantaneously and values are averaged per bee and written to an output file (Fig 1b).

**Table 2:** Overview of time penalties (time budgets) during the foraging cycle.

Behaviour	Time penalties for:	Duration (s)	Controlling variables
1a. Forage flowers in poor habitat	Assessing patch quality	1	
1b. Forage flowers in rich habitat	Flying to a flower	3, 4, 7	*medium velocity* (*body length*) and *flower density*
	Full flower: removing pollen from flower	30, 8, 2	*handling time* (*body length*)
	Empty flower: assessing flower	1	
2. Neighbouring cell	Accepting or denying surrounding cell	1	
	Flying to a surrounding cell	14, 21, 34	*medium velocity* (*body length*) and *landscape detail*
3. Fly around	Distance flown, per flight unit	5, 5, 5	*high velocity* and *length of flight units* (*body length*)
4. Fly back	Distance flown, per flight unit	5, 5, 5	*high velocity* and *length of flight units* (*body length*)
5. Nest reached	Pollen deposition	30	*time at the nest*

The minimum duration is one second only used for three penalties. Other penalties mostly relate to body size, giving three different values in those cases.

### 2.3. Verification, parameterization, validation and sensitivity

The model SOLBEE was verified (*sensu* [48]), parameterized and validated in a previous study [37] to which all statements in this section refer. The model produces natural foraging patterns (including an increase in foraging distances, foraging trip durations and flower visits per time unit during the foraging day as one would expect with depleted flowers around the nest) and realized foraging distances were much lower than general homing distances. Parameterization of the model input was mainly based on a literature review and included selection of appropriate values from within the ecological range (for details see [37]). The observed response variables overlapped well with independent literature values (other than used for input), which can be considered as a rough validation. However, model bees were found to be somewhat more efficient than real bees, yielding a higher number of brood cells, partly because the model omitted long lasting activities at the nest. Investigation of two main time budget-affecting parameters (*time at the nest* and *flytime*) revealed that the responses were not affected in their relative differences between bee types and landscapes. There can be strong effects of parameters when considering the complete ecological range, especially regarding vegetation parameters (flower pollen production and plant density) and landscape quality (regulating initial bee densities). Extreme parameter values helped understanding the model [37], but do not represent common vegetation (e.g. sunflower or clover monocultures would be such extremes) and extremely high and low bee densities and were not further considered. Additional simulation experiments showed that the most uncertain parameters did not have relevant effects on the responses and that all parameters could be considered robust against small changes.

### 2.4. Description of the simulation experiment

The aim of the simulation experiment was to gain mechanistic understanding of how communities of different solitary bee types (differing in nesting preference and body length) respond to landscape fragmentation in landscapes with different foraging habitat availability and provide pollination services at the local landscape scale. We generated one hundred different landscapes by varying *landscape fragmentation* (10 levels from 0.05 to 0.95 with equal intervals) in ten landscapes with different *foraging habitat availability* (proportion from 0.05 to 0.95). For each of these landscapes we let six different bee types (*body length* of 6, 12, and 24 mm combined with two *nesting preferences*, scenario-like) nest and forage for pollen in separate simulations. We simulated 20 replicates (20 give robust results and are a good trade-off between the cost of long simulation times and robustness of results, S2 Appendix Fig A) for each of the 100 landscapes and six bee types, a total of 12.000 simulations. To ensure that results are robust considering the vegetation parameters, we repeated these simulations with a different set of vegetation parameters (Table 1) with 5 replicates. This altered vegetation is characterized by a lower flower density (*fd* = 25) and larger flowers with a threefold of pollen per flower (*ppf* = 1.5), which is released in at least 5 pollen packages (*plimit* = 0.2).

### 2.5. Analysis of responses

We analysed the main response variables (brood cells, flower visits, visited foraging habitat and foraging distance) with R [49] with linear models, testing predictors and predictor interactions (including quadratic and cubic terms when necessary). A statistical analysis in a classical sense is not meaningful for a model where most processes are deterministic. P-Values for predictor and predictor interactions fall all below the 0.05 level and are therefore omitted. Instead, the sum of squares of an ANOVA was used to calculate the percentage explained by each predictor as well as the adjusted R^2^ to compare model fits.

First we investigate the effect of fragmentation in different landscapes (varying foraging habitat amount) on the responses, as well as the effect of nesting preference and body size. For a clearer understanding of the results we also investigate fragmentation-affected measures such as nest habitat availability, local bee density and the ratio of nest to foraging habitat. Each of these predictor variables is hypothesized as simplifying replacement of landscape fragmentation and foraging habitat availability and may ideally also simplify contrasts within bee traits.

## 3. Results

### 3.1. Response to fragmentation

**Fig 2:**
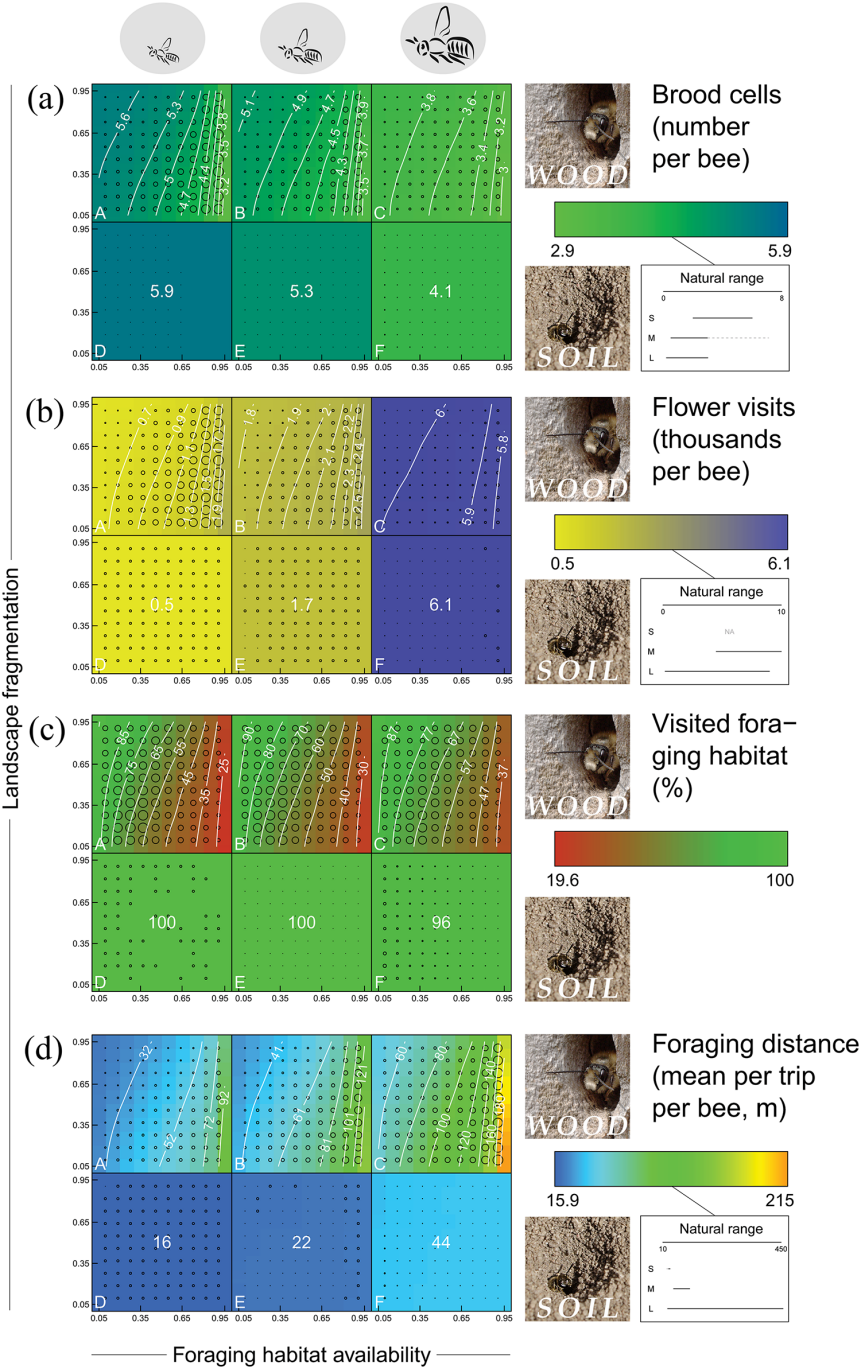
Simulated responses to landscape fragmentation in landscapes with different foraging habitat availability. Each model response, i.e. brood cells (a), flower visits (b), visited foraging habitat (c) and foraging distance (d), is displayed for six bee types (panels A-F) to illustrate the effect of traits. The panels depict from the left to the right small (panel A, D), intermediate (panel B, E) and large bees (panel C, F) and from top to bottom wood-nesting (panel A-C) and soil-nesting (panel D-F) bees. Within each panel 100 different simulated landscapes are displayed and characterized by a gradient of *landscape fragmentation* (bottom to top) and *foraging habitat availability* (left to right), with each landscape being represented by a single coloured square (legend on the right for each response, accompanied by natural ranges) being the mean of 20 replicate simulations. Contour lines visually guide the gradients that are present in these 100 means (calculated by prediction). The standard error to each mean of 20 replicates, as complementary information, is indicated by a circle. The smallest circle always represents zero, while the largest circle represents 0.09 for brood cells, 0.05 for flower visits, 2.29 for visited foraging habitat and 4.92 for foraging distance. Natural ranges (black lines and extreme values as grey dotted line) were reviewed and discussed (such values are rarely measured and not well known for bees of different size) in an earlier manuscript ([37]) and are added here for rough comparison with the results only (as the model's aim is to understand the system and not to reproduce exact values). Photographic material by the main author, also available on Wikimedia.

The performance of a simulated bee was measured in terms of number of brood cells, number of flower visits, percentage visited foraging habitat and mean foraging distance, based on 20 replicate simulations. *Landscape fragmentation* had a small positive effect on the number of brood cells (Fig 2a) and visited foraging habitat (Fig 2c) and a negative effect on foraging distance (Fig 2d, Table 3). In landscapes with more foraging habitat, the number of brood cells and the percentage visited foraging habitat was lower (Fig 2a and 2c) and foraging distance higher (Fig 2d). *Landscape fragmentation* and *foraging habitat availability* were far less important than *body length* and *nesting preference* (Table 3) and the interaction between *landscape fragmentation* and *foraging habitat availability* explains only little of the observed variation in bee performance.

**Table 3:** Analysis of variance for the number of brood cells, flower visits, visited foraging habitat and foraging distance in response to landscape fragmentation.

	Response							
Predictors	Brood cells		Flower visits		Visited foraging habitat		Foraging distance	
	% Explained	F value	% Explained	F value	% Explained	F value	% Explained	F value
landscape fragmentation	0.5	1138.4	0.0	288.1	1.1	3277.5	0.9	1405.2
foraging habitat availability	5.8	14014.1	0.2	4986.8	14.5	42299.9	14.3	21208.5
body length	64.0	77139.3	98.4	1582381.0	0.1	123.7	21.0	15580.0
nesting preference	16.2	39080.9	0.3	10344.7	63.4	185096.4	37.6	55667.2
landscape fragmentation × foraging habitat availability	0.1	129.5	0.0	55.0	0.0	5.3	0.1	81.2
landscape fragmentation × body length	0.1	69.4	0.0	203.6	0.0	22.8	0.1	39.2
landscape fragmentation × nesting preference	0.5	1141.3	0.0	286.4	1.1	3290.2	0.9	1316.8
foraging habitat availability × body length	0.7	903.0	0.2	2817.4	0.2	241.9	1.2	917.6
foraging habitat availability × nesting preference	5.9	14317.0	0.2	4879.4	14.6	42662.5	13.6	20139.9
body length × nesting preference	1.2	1421.5	0.4	6653.5	0.8	1173.2	2.2	1639.6
Residuals	5		0.4		4.1		8.1	
*df used / residual df*	*14*	*11985*	*14*	*11985*	*14*	*11985*	*14*	*11985*
*(adjusted) R* ^*2*^	*0*.*95*		*1*.*00*		*0*.*96*		*0*.*92*	

The analysis is based on 20 replicates (12,000 simulations). Additional predictors were landscape type (foraging habitat availability), the bees' body length and nesting preference and all interactions. Given are F-value, % explained, the used degrees of freedom (used df), remaining degrees of freedom (residual df) and adjusted R^2^. The × indicates an interaction between two parameters. Further details are given in the methods.

### 3.2. Trait effects

*Body length* explained more than 98% of the variance of flower visits, compared to less than 1% for *nesting preference* (Table 3). *Body length* explained less than 1% of the variance of visited foraging habitat, while *nesting preference* explained over 63% (Table 3), being almost opposite to flower visits. Large bees visited most flowers (Fig 2b) and soil-nesting bees covered most foraging habitat (Fig 2c). Nesting preference clearly affected the way bees respond in different landscapes (interaction of *nesting preference* with *foraging habitat availability* and with *landscape fragmentation*, Table 3), with soil-nesting bees having a similar response in all landscapes (Fig 2) and wood-nesting bees were responding to *foraging habitat availability* and *landscape fragmentation* as described in the following. Brood cells and foraging distance were affected by both traits (Table 3). The number of brood cells was highest for small, soil-nesting bees (Fig 2a). In contrast, foraging distance was highest for large, wood-nesting bees (Fig 2d). Within a given *body-length* category, mean foraging distance and mean number of brood cells were highly correlated (*r*^*2*^ = 0.998).

### 3.3. Nest habitat, local density and relative habitat availability

**Fig 3:**
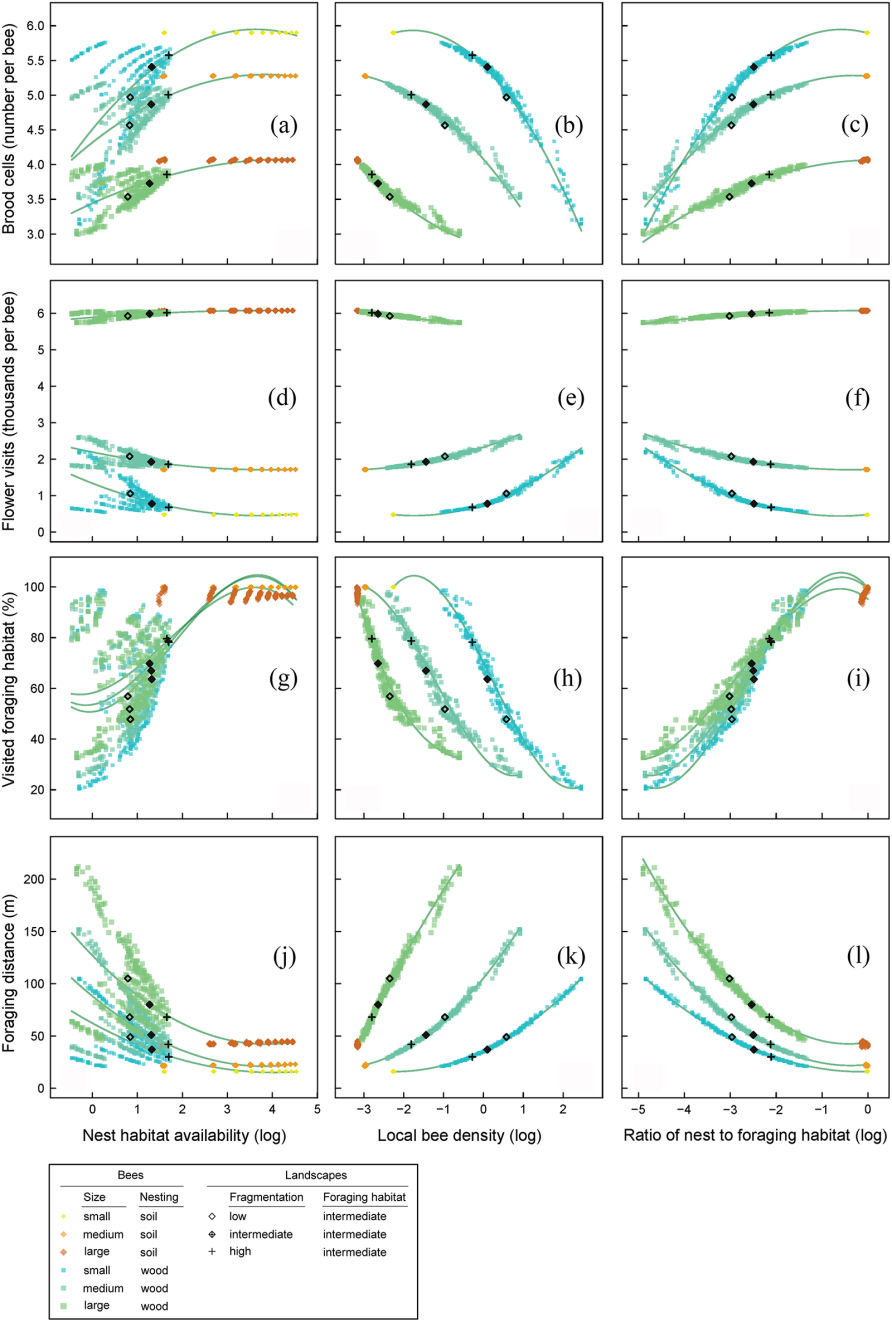
The simulation results in response to single landscape-level gradients. The simulation results for the four responses (rows)brood cells (a-c), flower visits (d-f), visited foraging habitat (g-i) and foraging distance (j-l) in response to three landscape-level gradients (columns) of: nest habitat availability (left column, a,d,g,j), local bee density (middle column, b,e,h,k) and the ratio of nest to foraging habitat (right column, c,f,i,l). The three y-axes in each row have equal dimensions and are labelled on the most left figure of each row, while the four x-axes in each column have equal dimensions as well and are labelled on the bottom figure of each column. Points are plotted for three replicate simulations (for 100 landscapes) with six colours representing the different bee types (i.e. 1800 points per plot). Lines are based on the full linear models with 20 replicate simulations. How landscape fragmentation affects each gradient is visualized with a landscape with intermediate *foraging habitat availability* (0.45) increasing from low *landscape fragmentation* (0.05) to intermediate (0.45) to high (0.95) for small, intermediate sized and large wood-nesting bees (i.e. nine points of 1800 are plotted with extra symbol).

Landscape-level predictors (*nest habitat availability*, *local bee density* and the *ratio of nest to foraging habitat*) each predicted more (Table 4) than *landscape fragmentation* per se (Table 3). Local bee density and the *ratio of nest to foraging habitat* could, in combination with body length (and without *nesting preference*), well explain brood cells, flower visits, visited foraging habitat and foraging distance (adjusted R^2^ near to 1, Table 4), while *nest-habitat availability* had lower explanatory power (Table 4). The number of brood cells (Fig 3a and 3c) and visited foraging habitat (Fig 3g and 3i) increased with *nest habitat availability* and with the *ratio of nest to foraging habitat*, while *local bee density* increased foraging distance (Fig 3k) and conditionally flower visits (Fig 3e, small and intermediate sized bees).

**Table 4:** Analysis of variance for the number of brood cells, flower visits, visited foraging habitat and foraging distance (top to bottom) in response to one of three focal predictors; nest habitat availability, local bee density and ratio of nest to foraging habitat (left to right) based on 20 replicates.

		Focal predictor					
Response	Predictors	1. Nest habitat availability (log)		2. Local bee density (log)		3. Ratio of nest to foraging habitat (log)	
		% Explained	F value	% Explained	F value	% Explained	F value
Brood cells	Body length	64.0	23859.1	64.0	860482.0	64.0	840299.0
	Focal predictor	16.1	11977.6	31.1	835541.7	27.6	724097.8
	Focal predictor × body length	1.9	1433.6	1.3	35845.7	4.2	111497.2
	Focal predictor ^2^	1.4	537.9	2.3	30834.5	2.6	34720.1
	Focal predictor ^2^ × body length	0.4	152.8	0.8	10450.4	1.0	13132.7
	Residuals	16.1		0.4		0.5	
	*df used / residual df*	*8*	*11991*	*8*	*11991*	*8*	*11991*
	*(adjusted) R* ^*2*^	*0*.*84*		*1*.*00*		*1*.*00*	
Flower visits	Body length	98.4	754615.1	98.4	32600248.0	98.4	32762139.0
	Focal predictor	0.3	4949.9	1.0	679119.5	0.6	393985.7
	Focal predictor × body length	0.0	511.0	0.4	282254.9	0.1	69555.9
	Focal predictor ^2^	0.4	3261.9	0.1	40831.5	0.8	251784.1
	Focal predictor ^2^ × body length	0.1	452.8	0.0	9936.9	0.2	50569.7
	Residuals	0.8		0		0	
	*df used / residual df*	*8*	*11991*	*8*	*11991*	*8*	*11991*
	*(adjusted) R* ^*2*^	*0*.*99*		*1*.*00*		*1*.*00*	
Visited foraging habitat	Body length	0.1	12.8	0.1	326.9	0.1	340.5
	Focal predictor	54.5	16385.8	90.0	694258.9	92.0	739233.4
	Focal predictor × body length	1.1	340.3	1.2	9455.5	2.5	20004.1
	Focal predictor ^2^	0.8	121.9	1.5	5931.2	1.5	5831.4
	Focal predictor ^2^ × body length	0.0	0.4	3.6	13972.2	0.0	18.8
	Focal predictor ^3^	3.6	1073.7	1.8	13945.4	2.3	18666.9
	Focal predictor ^3^ × body length	0.1	11.4	0.1	495.4	0.1	420.9
	Residuals	39.9		1.6		1.5	
	*df used / residual df*	*11*	*11988*	*11*	*11988*	*11*	*11988*
	*(adjusted) R* ^*2*^	*0*.*60*		*0*.*98*		*0*.*99*	
Foraging distance	Body length	21.0	3444.1	21.0	195465.8	21.0	182885.1
	Focal predictor	35.8	11716.2	51.9	965066.7	63.4	1102137.0
	Focal predictor × body length	4.5	1464.0	5.0	92574.0	10.4	180286.2
	Focal predictor ^2^	1.8	299.7	20.9	194541.9	3.7	31869.9
	Focal predictor ^2^ × body length	0.3	46.4	0.5	4560.2	0.9	7603.8
	Residuals	36.6		0.6		0.7	
	*df used / residual df*	*8*	*11991*	*8*	*11991*	*8*	*11991*
	*(adjusted) R* ^*2*^	*0*.*63*		*0*.*99*		*0*.*99*	*0*.*99*

The layout of these twelve linear models follows the layout of the plots in Fig 3. Body length is included as co-predictor, as well as quadratic terms (and cubic for visited foraging habitat) and their interactions (with × indicated). Given are F-value and % explained. The used degrees of freedom (used df), remaining degrees of freedom (residual df) and adjusted R^2^ are given additionally for each model in *italic*. Further details are given in the methods.

Wood-nesting bees were restricted to edges for nesting, with nest habitat availability increasing with fragmentation, while soil-nesting bees could use large areas of the foraging habitat for nesting in the soil, always having a high *nest habitat availability* (Fig 3a, 3d, 3g and 3j). In a similar way, soil-nesting bees had the lowest *local bee density* and the highest *ratio of nest to foraging habitat*. *Body length* retained its effect, being strongest for flower visits (Fig 3d–3f) and weakest for visited foraging habitat (Fig 3g and 3h).

We exemplified the direction of the effect of *landscape fragmentation* on each of these landscape-level predictors and the responses for three selected landscapes (Fig 3). Fragmentation did not only result in more brood cells, fewer flower visits, more visited foraging habitat and shorter foraging distances, it also increased *nest habitat availability* and the *ratio of nest to foraging habitat availability* and reduced *local bee density* (Fig 3).

### 3.4. Altered vegetation, adjusted fitting and cross-relationships

The analysis was repeated for a different set of vegetation parameters (i.e. larger flowers in lower density), while all other conditions and methods remained the same (S3 Appendix). Changes in flower size and density led to a clear change in the relative importance of traits and fragmentation on the responses. The relative importance of *nesting preference* and *landscape fragmentation* were here higher for the number of brood cells but lower for visited foraging habitat (S3 Appendix Table A). In contrast, the relative importance of *body length* was lower for brood cells but higher for visited foraging habitat (S3 Appendix Table A). The changed effect of fragmentation is more prominent when the landscape-level predictors are concerned; the importance of *nest habitat availability*, *local bee density* and the *ratio of nest to foraging habitat* were especially higher for brood cells (S3 Appendix Table B).

**Fig 4:**
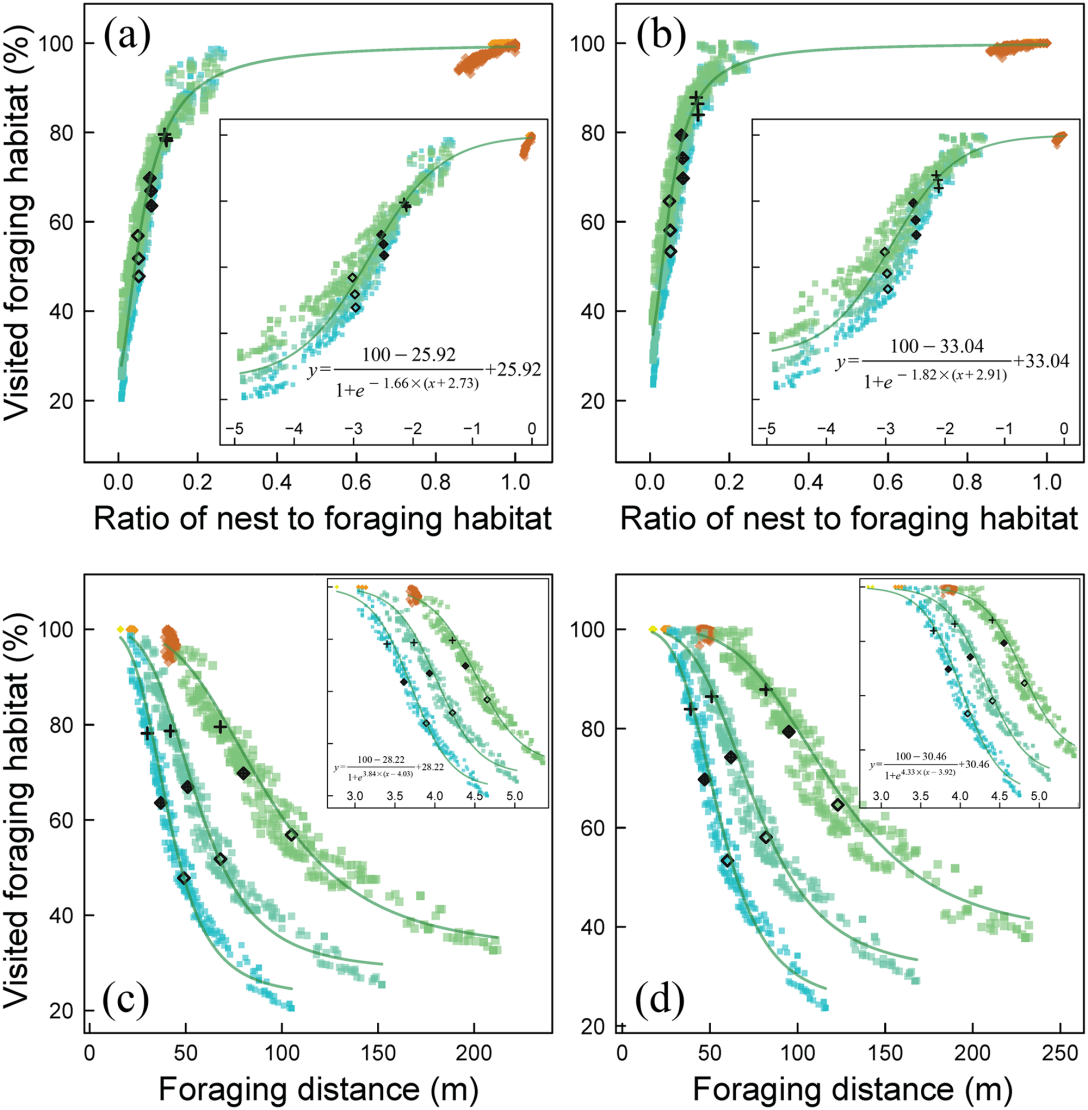
Comparison of two vegetation types for their effect on visited foraging habitat and for two conflicting pollination proxies both fitted with logistic functions. Visited foraging habitat in response to the ratio of nest to foraging habitat is plotted for a) initial vegetation (same conditions as in Fig 3i) and for b) with adjusted vegetation parameters. The logistic curves are fitted for all bees independent of nesting preference and body length (inlay with fitted values) and untransformed to the main plot. Two conflicting response variables, visited foraging habitat and foraging distance, are plotted against each other for: c) initial vegetation and for d) with adjusted vegetation parameters. The logistic curves are fitted for each body size separately (inlay with fitted values for intermediate sized bees) and untransformed to the main plot. Details for all curves are given in Table 5. All remaining conditions are the same as described for Fig 3.

Only one response was just slightly affected by bee traits over the complete analysis: the percentage visited foraging habitat. Therefore it makes sense to simplify and describe its response to the *ratio of nest to foraging habitat* (Fig 3i) with a single logistic curve (Fig 4a, repeated for the second set of vegetation parameters Fig 4b). The R^2^ values for both logistic models are hardly reduced when omitting *body length* (Table 5). This despite the fact that the second set of vegetation parameters led to a general increase in the relative importance of *body length*. The response curve of visited foraging habitat to the non-transformed *ratio of nest to foraging habitat* (Fig 4a and 4b) is notable for its almost linear increase which reaches almost 100% at a ratio of 0.2 (20% nest habitat compared to foraging habitat).

**Table 5:** Logistic fits for the relation between visited foraging habitat and the ratio of nest to foraging habitat for two vegetation types and the relation between visited foraging habitat and foraging distance.

Response	Predictor	Vegetation type	Corresponding figure	Body size (mm)	a	b	c	R^2^
Visited foraging habitat	ratio of nest to foraging habitat (log)	A	4a	all in one	1.66	-2.73	25.92	0.97
Visited foraging habitat	ratio of nest to foraging habitat (log)	B	4b	all in one	1.82	-2.91	33.04	0.95
Visited foraging habitat	Foraging distance (log)	A	4c	24	-3.54	4.52	32.04	0.98
Visited foraging habitat	Foraging distance (log)	A	4c	12	-3.84	4.03	28.22	0.99
Visited foraging habitat	Foraging distance (log)	A	4c	6	-4.03	3.71	22.88	0.99
Visited foraging habitat	Foraging distance (log)	B	4d	24	-4.04	4.79	37.63	0.97
Visited foraging habitat	Foraging distance (log)	B	4d	12	-3.92	4.33	30.46	0.98
Visited foraging habitat	Foraging distance (log)	B	4d	6	-4.13	4.02	24.08	0.99

Given are two vegetation types (A: initial vegetation and B: adjusted vegetation). Logistic functions follow the following format: f(x) = (100-c)/(1+*e*^-a·(x-b)^) + c. For each fit the parameters a, b and c are given and the accompanying adjusted R^2^. All curves are plotted in Fig 4.

Another finding, that the responses visited foraging habitat and foraging distance always responded oppositely (Fig 3g–3i compared to Fig 3j–3l), hints to a trade-off. They correlate negatively (Fig 4c and 4d) and non-linear (Table 5). With increasing foraging distance (or despite increasing, at first logical sight), the percentage visited foraging habitat decreases.

## 4. Discussion

In all simulations both large and small bees could, based on resource availability at the landscape scale, build the same number of brood cells and cover the complete foraging habitat. Thus, all observed differences in brood cell numbers (and pollination proxies) between soil- and wood-nesting bees and bees of different size is due to the different allocation of foraging and nesting resources, especially the latter being affected by fragmentation. The limited amount of time (a day) and the time budgets related to body size have resulted in differences.

### 4.1. Effects of spatial resource distribution

#### 4.1.1. Fragmentation effects on fitness

By design, all bees had the opportunity to build the same number of brood cells within a day assuming spatial allocation of nests and pollen producing flowers would not play a major role. This was indeed the case for soil-nesting bees of the same size, which did not respond to *landscape fragmentation* (Fig 2a) or to the ratio of nest to foraging habitat (Fig 3c). They had optimal access to foraging resources from their nest, because nests were distributed randomly over the foraging habitat (Fig 1c). Wood-nesting bees, in contrast, responded positively to *landscape fragmentation*. This supports the hypothesis that fragmented landscapes increase bees diversity by providing increased nest-site availability [26, 50], which applies to wood-nesting bees restricted to edge habitat. In our model, bees were indeed positively affected by nest-habitat availability (Fig 3a). Although the interaction effect between *foraging habitat availability* and *landscape fragmentation* was low, wood-nesting bees seem to respond stronger in landscapes with high *foraging habitat availability* (Fig 2a).

The apparent decrease in brood cell number with increasing *foraging habitat availability* (Fig 2a) is a side-effect of the model setup, highlighting the importance of time and time-budgets. The number of individuals per landscape was by definition linked to *foraging habitat availability*. The resulting local bee density (and hence competition around nest sites for resources) had a strong negative effect on brood cell number (Fig 3b). This supports the idea that time can be more limiting for wild bees than foraging resources [47]. Spatially induced time constraints deserve more attention in pollinator research. It can for example be argued that oligolectic and monolectic bees (highly specialized on certain plant species, e.g. Andrena hattorfiana [51]) face very low plant densities at the landscape scale and have to deal with strong time constraints, an additional competitive disadvantage compared to polylectic bees.

We showed that traits (*nesting preference* and *body length*) affect the response to fragmentation, explaining why some studies find no effects of fragmentation on bee performance [20, 26], while others did [19, 27]. Lack of response to fragmentation in field studies could relate to dominance of certain traits in the community (e.g. a large proportion of bumblebees). Nevertheless, we also recognize that the effect of *landscape fragmentation* can be low compared to other landscape-level parameters (compare Tables 3 and 4) and may remain undetectable in many field studies, suggesting a need for alternative measures. Finally, also an increase in *foraging habitat availability* above an intermediate amount reduced the amount of edge structures (merging of patches), which is independent of *landscape fragmentation* (e.g. nest habitat availability is frequently low beyond low *landscape fragmentation*, Fig 3a), again demonstrating that *landscape fragmentation* on its own is a poor measure.

#### 4.1.2. Fragmentation effects on pollination

The pollination measures (number of flower visits, percentage visited foraging habitat and mean foraging distance) largely followed the patterns for the number of brood cells. Pollination by soil-nesting bees remained largely unaffected by fragmentation. However, the pollination performance of wood-nesting bees was affected by fragmentation. Wood-nesting bees visited fewer flowers (Fig 2b, small and intermediate sized wood-nesting bees) and flew shorter distances (Fig 2e) with increasing *landscape fragmentation*. Under high local bee density (low *landscape fragmentation*), bees inevitably encountered empty flowers more frequently and were forced to visit and probe more flowers (Fig 3e) and fly longer distances (Fig 3k). This means that fragmentation can both reduce the frequency of pollen transfer between flowers and reduce the mean distance over which the pollen is transferred. *Landscape fragmentation* increased the landscape-level coverage with pollinators (percentage visited foraging habitat, Fig 2c) by a better distribution of nest habitat over the landscape.

#### 4.1.3. Linking fragmentation, nest habitat, bee density and the ratio of nest to foraging habitat

Increased *landscape fragmentation* resulted in higher nest availability, a lower local bee density and a higher ratio of nest to foraging habitat. Local bee density and the ratio of nest to foraging habitat were both better predictors for brood cells, visited foraging habitat and foraging distance than *landscape fragmentation* itself. The nest to foraging habitat seems a suitable landscape-level parameter to replace *landscape fragmentation*, *foraging habitat availability* and *nesting preference* by a single measure, giving an informative gradient of habitat complementarity for wild bees [11], capturing local landscape quality well (Fig 3c, 3f, 3i and 3l). Although local bee density was an equally good predictor—and can in principle be measured in the field—it is less practicable because it requires high sampling effort. The ratio is probably easier to approximate with local information about potential bee habitats.

The result that bees need an appropriate ratio of nest habitat relative to foraging habitat can be considered a novel, but also logical, insight gained by modelling practice. We believe that the general observation that wild bee communities are mainly affected by foraging habitat availability [18–20] is therefore incomplete. The hypothesis that solitary bees are most limited by nest sites [20–22] applies therefore, according to our results, only when foraging resources are constant. The here proposed ratio of nest to foraging habitat availability could satisfy the need for a landscape measure that is more suitable in describing the resource needs of solitary bees [36].

### 4.2. Effect of traits on spatial foraging

#### 4.2.1. Body size

One would expect a large bee to visit more flowers (required for a full load) and fly longer distances than a small bee. This was in general true (Fig 2b and 2d). As a result, large bees built fewer brood cells than small bees, which is also supported by field observations (reviewed in [37]). The expectation that large bees perform better than small bees in fragmented landscapes because of their faster movement [50] and shorter flower handling times, turns out to be wrong, at least under the assumptions made.

The number of visited flowers was the response most affected by *body length*. Obviously, large bees need to visit more flowers for their pollen requirements than small bees (Fig 2b), in accordance with field studies [52, 53]. The difference in flower requirements per brood cell between large and small bees is even higher than the difference in daily requirements, given that large bees also build fewer brood cells. Time allocation diagrams for the different bees (S2 Appendix Fig B) make clear that model bees spent far more time visiting flowers than for flying between them or back to the nest. Therefore, flower-handling time is the most important trait for compensating high pollen requirements. This trait was in the sensitivity analysis indeed the most influential one among other size-related traits [37].

When flowers are larger, the explained variance by *body length* for brood cells drops more than half, the most prominent difference for altered vegetation parameters (S3 Appendix Table A). Larger flowers bring an advantage for large bees when they do not have to visit so much more flowers than smaller bees (indeed reduced explained variance by *body length* for flower visits, S3 Appendix Table A).

Small bees have an advantage compared to large bees, but they are also more sensitive to changes in the landscape. Small and intermediate-sized bees (wood nesting) visited fewer flowers with increasing *landscape fragmentation*, while large bees (wood nesting) visited more flowers (Fig 2b). In addition, the crossing lines in Fig 3c show that there are also landscapes in which small bees perform worse than larger bees. In landscapes with much foraging habitat and little nest habitat (low nest to foraging habitat ratio, Fig 3c) local nest density of bees is very high with small bees having the highest nest densities (Fig 3b). This nest density problem is most problematic for small bees due to the mass proportional relation between body size and bee numbers in the landscape (S1 Appendix Table D). At some point the density of small bees becomes so large that flying farther from the nest for foraging resources (Fig 3k) cannot compensate this, leading to a very steep decrease of brood cell number which is absent for large bees (Fig 3b). Also does an increase in local bee density imply more visits of empty flowers, causing an increase of total flower visits for small bees, while large bees show a slight decrease (Fig 2e), explaining the different responses to *landscape fragmentation* (which reduces local bee density). Large bees seem to escape local overpopulation more easily (steeper increase in mean foraging distance with local bee density, Fig 3k). Consequently, especially small bees could be driven to evolve mechanisms for optimizing their foraging behaviour and to quickly sense high local bee densities and empty flowers (e.g. by smart flower probing rules), to justify the investment in farther flights.

Larger bees seem in general to be better pollinators: they visit more flowers, cover more foraging habitat and fly longer distances. For visited foraging habitat is the effect of body size lowest, and small bees cover the foraging habitat almost as much as large bees (Fig 3g–3i) although they fly on average less far from the nest (Fig 3j–3l). The pollination potential of small insects is often underestimated and they transfer enough pollen for sufficient seed set [54].

#### 4.2.2. Nesting preference

*Nesting preference* was the most important predictor for foraging distance and for visited foraging habitat and second important for the number of brood cells. Soil-nesting bees were evenly distributed over the foraging habitat and found foraging resources near the nest, leading to short foraging distances (Fig 2d) and optimal coverage of the foraging habitat (Fig 2c). The maximum number of brood cells within a day (Fig 2a) was hardly affected by the gradient of *landscape fragmentation*. Wood-nesting bees, in contrast, responded strongly to different landscapes. They had a lower brood cell number in landscapes with a low degree of *landscape fragmentation* (Fig 2a) where they had longer foraging distances (Fig 2d). This makes sense in relation to lower nest habitat availability (Fig 3a), higher local bee density (Fig 3b) and low ratio of nest to foraging habitat availability (Fig 3c). Wood-nesting bees also covered less foraging habitat when there was more foraging habitat to cover in the landscape (down as low as 25% for high *foraging habitat availability*, Fig 2c). Bees nesting at field edges did not reach the interior of the fields in those cases. *Nesting preference* hardly affected the number of flower visits (Fig 2b), which seems rather being affected by body-size related traits alone.

Soil and wood nesting is often considered as a factorial contrast between bees, but the model shows otherwise. There seems to be a gradient of habitat use, where both nesting preferences are part of the same relationship for a bee of a certain size (Fig 3, all panels). Most models explained the variance sufficiently without *nesting preference* (Table 4). Soil-nesting bees in natural bee communities often nest in very high densities [55] and occur in much higher numbers on fallow land than wood-nesting bees [19], suggesting that soil-nesting should be modelled more restricted in nest habitat availability. This would likely complete the visible gap (especially in the ratio of nest to foraging habitat, Fig 3c, 3f, 3i and 3l).

### 4.3. Pollination considerations

#### 4.3.1. Trade-offs between pollination measures

We found a trade-off between mean foraging distance and coverage of the habitat with pollinators at the landscape level (negative relationship, Fig 4c and 4d). This means that either pollen is transported over larger distance or that pollen is transported at all places in the foraging habitat, not both. In the best landscapes for bees (e.g. high ratio of nest to foraging habitat), bees were spatially optimally distributed leading to a high percentage of visited foraging habitat (Fig 3i), also leading low mean foraging distances (Fig 3l). Oppositely in bad landscapes, bees are forced to fly farther distances, but do not cover all foraging habitat remaining certain areas unpollinated. In agricultural landscapes it is common that bees are most abundant in field edges [56, 57]. Field interiors often show low abundance of solitary bees, and bees foraging there may be soil-nesting bees provided locally with nests [58].

#### 4.3.2. Up to 20% suitable as nesting habitat

The simulation results suggest that visited foraging habitat (coverage with bees) increases steeply up to a nest to foraging habitat ratio of 0.2 (i.e. 20% nest habitat compared to foraging habitat) and beyond this levels off to optimal coverage (close to 100%, Fig 4a). The value seems robust for vegetation type (Fig 4a and 4b) and bee type. It may suggest a general pattern applying to bees as pollinators in general, but needs deeper investigation and field validation for broader application. This value reminds of the recommendation that about 25% of the landscape should remain refugee area for wild bees to maintain sustainability [59]. A recent review [60] estimates that 2 to 44% of the landscape with high quality flower strips is required for the sustainability of pollinator communities. However, those numbers focus on a different aspect of the bees' ecology (sustainable populations) and do not consider the required amount of nest habitat (and ratio of bee habitats) to pollinate a certain area. Hence, data-based estimations of the optimal ratio remain a future concern.

A simple calculation illustrates that most agriculturally dominated landscapes are really poor and a hostile environment for solitary bees, despite mass flowering crops. For example, providing a one ha crop field with a five-meter wide natural strip yields a desired ratio of 0.19. The same five-meter strip at a 49 ha field yields a ratio of 0.03. Practically considered, to improve the local ratio, the nest habitat for bees should be sufficient (increase of unmanaged field strips) of good quality (reduced pesticide application) and spatially well distributed within an area (fragmented spatial distribution). We recognize that 20% field strips is not economically feasible in modern agriculture, but we agree with others that management encouraging a mosaic of smaller fields and increase of the edge:area ratio would benefit pollinators [61]. For example, up to 8% of the field edge can be converted to bee-friendly habitat without losing yields [62], because the positive effect on pollinators (increased pollination) compensates for the smaller crop area.

In situations where the ratio remains far below 0.2 (e.g. large crop fields), a solution may be the provision of artificial nest sites. There are many examples where solitary cavity-nesting bees are employed as crop pollinators, by offering artificial nests [63, 64]. Also, at least one soil-nesting species (*Nomia melanderi*) is managed as crop pollinator by offering nesting beds in the soil [65, 66].

### 4.4. General considerations

#### 4.4.1. Foraging distances

The mean foraging distance from the nest is a measure for how far pollen is transported and it is focused on the motivation of the bee. Assuming that bees do not fly farther than necessary can result in very short foraging distances for soil-nesting bees (< 50 m, Fig 2d) in accordance with natural ranges from field studies [37]. Their nests were evenly distributed over the foraging habitat and they found enough pollen within a short range from the nest in all landscapes. Mean foraging distances are higher when conditions are unfavourable, e.g. when bees face a high local nest and bee density in field edges (wood-nesting bees) and untouched foraging resources only farther away. Large bees flew farther (50–200 m) than small bees (30–100 m). These still relatively low distances are in agreement with the finding that that both large and small bees often forage below 200 m [67]. Some studies may overestimate foraging distances, especially when solitary and eusocial bees are not separated or landscape types are not considered [16]. Honeybees and bumblebees fly farther, as well as solitary bees without any foraging recourse near the nest. The mean foraging distance as measure for pollination does not provide information on extreme events (beyond the mean) that may lead to pollination as well. Nevertheless, it is clear that bees mainly exchange pollen between plants over short distances and that most activity is near the nest.

#### 4.4.2. Matrix crossing

Short foraging distances imply that bees did not often fly to more distance patches and did not often cross the matrix without foraging resources, despite a parameter that induces such behaviour. Matrix crossing behaviour was modulated by the model parameter *ignorance* (Table 1), which was earlier shown not to affect the model simulations much [37]. Low matrix crossing behaviour is in accordance with the idea that wild bees live on islands of foraging habitat [9] and is reported for various pollinators [68, 69]. Solitary bees have a high site fidelity with very conservative movement patterns [70], which does not seem to be improved by grassy field strip corridors [57]. The idea that solitary bees, as flying insects, often display matrix crossing behaviour and fly long distances needs to be further refined by future field and modelling studies. In ecosystem-service research, such concepts are trivial for estimating the spatial availability of pollination services.

#### 4.4.3. Size and evolution

The result that large solitary bees are on average worse performers than small ones may imply evolutionary consequences. The lower efficiency of large bees may be a driver to develop a social structure with higher efficiency and explain why large bees are more often social than small bees. In central Europe there are e.g. more species of eusocial bumblebees than large bees from the genus *Xylocopa* or *Anthophora*, in contrast to very small bees which are often solitary (e.g. *Andrena*) or only primitively eusocial (e.g. *Lasioglossum*). The performance constraint may thus be an additional driver for sociality in combination with other evolutionary drivers such as climate change [71] and time (lineage age [72]). At the same time it may explain why in regions where bees have been under pressure for decades, body size decreased over time at the species level [73].

#### 4.4.4. Ratio of habitats as simplified measure

Foraging habitat visitation increased with the ratio of nest to foraging habitat with a remarkably low effect of *body length*. This ratio seems a robust proxy descriptor for landscape structure, adapted to the perspective of bees and suitable for estimating pollination services. We expect that the ratio of nest to foraging habitat, after further study, can become an easy to calculate landscape simplification in field studies and a valuable addition to other indexes such as the LLI [74, 75]. When more field surveys include the identification of nest habitat in addition to foraging habitat, we expect that the positive effects of the ratio can be soon be confirmed.

### 4.5. Future considerations

#### 4.5.1. Notes on biological assumptions in the model

The combined fact that we tested all model parameters [37], that the model's output values come close to values from real systems (Fig 3), and that we ensured that our results hold for altered vegetation parameters, confirms that we have successfully covered a part of a complex multifactorial system and understand this part a little better. As with most models, some biological assumptions lie in the intrinsic structure of the model itself and are not critically tested, while at the same time simplifications are inevitable to speed up calculation. Some of our decisions and considerations should be mentioned for future model implementations. One example is the unrealistically high number of brood cells, which can easily be justified but also criticized. We assumed a short *time at the nest* after each trip and we neglected time needed for egg laying, cell closure and other activities (due to data deficiency and unnecessary complexity, see also [37] for a discussion). We further assumed that all pollen collected at the flowers reach the nest, while in reality pollen gets lost on the way [76] and solitary bees of the same species return to the nest with a high variability of pollen loads [77]. Also, in favourable landscapes bees may be egg-limited rather than pollen limited [38]. Some assumptions may have excluded important mechanisms from the model, such as the assumption that bees also fuel themselves with nectar on the same flowers when they forage for pollen. The time budget for nectar collection is relatively small compared to that for pollen collection (see [37]) and may be negligible, but we do not know the effect of reducing a decision sequence depending on two resource levels to a single one. Finally, considering the many different parameters that were parameterized with heterogeneous sources, the model may benefit systematic studies for each parameter to improve accuracy. To identify priorities, a future study could address how changes in the allometric rules affect time budgets (Table 2) and identify the rule requiring the highest quality data. Also the effect of potential correlations, such as a positive relation between body size and flower size preference (which probably would minimize the disadvantage for large bees that we found) or a positive relation between body size and *time at the nest*, could be of future interest, even when studies failed to prove a systematic difference in flower preference [78, 79] or *time at the nest* (reviewed in [37]) so far. In the end, the priory of considering such issues depends on the questions for which the model is used.

#### 4.5.2. Towards simulation of real communities

We chose to focus on fragmentation, kept other parameters constant and compared six bee types in a scenario-like way to reduce complexity and understand model processes. As may be desired by field ecologist working with pollinators, simulation of realistic plant and bee communities requires a data-driven approach, in which values are parameterized and combined in sets. For a simulation with multiple bee species one needs community data on species composition, local bee densities and nesting locations. It is already challenging to get such sufficiently detailed data in the field, but the model also requires definition of flower traits making up the vegetation, including pollen provision and flower density. As a minimum, the distribution of these traits in a community is needed to simulate realistic vegetation patches. At the same time, additional model rules may be required when bee species face different types of flowers or when the presence of other species affects foraging behaviour [80] or brood cell number [81]. A further advantage of such an attempt with real bee densities for each species separately is that it overrules the assumption that pollinator density scales negatively with body size and positively with foraging resources, which had a prominent effect on our results.

#### 4.5.3. Applications with crop fields and semi-natural edge habitats

The model system may roughly represent crop systems: fields with foraging resources and edge habitat with nesting resources for wood-nesting bees (and in some cases suitable soil within the crop field). However each crop has different properties that are not necessarily covered by our current simulations. Flower size (pollen production per flower) and flower density can be much more extreme, such as a clover field with very small flowers in high densities or a sunflower field with very large flowers in low densities. We did not model this explicitly for several reasons. We focussed on a more general system to understand wild bees as pollinators and general patterns, but specific crops systems could be focus of future studies. The current results help to think in terms of flower sizes and densities in real systems and to interpret other studies in this light. For example, in a recent study wild bees were hardly found in sunflower field interiors [82], which may be a result of enough pollen being offered near the nest, explaining that the quality of the edge habitat did not have an effect on the pollinator community in the interior of the field [82]. Our model suggests that an increase in fragmentation and the ratio of nest to foraging habitat at a very local scale should increase pollinator coverage largely independent of flower size.

A different challenge for the future is to see and treat an agricultural field as potential nest habitat for wild bees. When soil-nesting bees are limited to field edges they must fly farther distances from the nest and face more local competition with bees around the nest. In general, soil disturbance in crop fields is assumed to be too high for soil-nesting bees to survive [83–85], but recent studies suggest that it is possible [58, 86] for species that nest a meter below the surface. Agricultural practices that reduce the depth of mechanical 'action' and pesticide application will benefit the survival of soil-nesting bees within fields. This would give an optimal nest to foraging habitat ratio and lead to better pollination.

#### 4.5.4. Combining strengths of pollinator models

Several models with foraging bees in response to vegetation seem to have being developed in parallel [37, 87–90]. Pollination ecology and agricultural ecology could benefit from a kind of "master-model" in which strengths are combined. This requires important decisions on how to deal with different scales (requiring different model elements) and reduce complexity in favour of simulation time. We think that some elements in our model that are unpractical in application can be simplified. For example, our decision to use mass-scaled resource availability for each bee (resulting in many small bees being compared to a few large bees), is not very practical to combine with an IBM approach, since a high variance in individual numbers also results in a high variation in calculation times. Also, some of our parameters are elaborate to measure and may benefit clever proxies. Presently, each model has its own advantages and level of detail, applicable to selected research questions.

### 4.6. Conclusions

The model applied in this study is a resource competition model at the time scale of one day, which measures performance parameters at the bee level as proxies for fitness and pollination. Model simulations showed that fragmentation of foraging habitat patches had positive effects on wood-nesting bees, but not on soil-nesting bees. Wood-nesting bees nesting in field edges clump to higher local nest densities and profit from a higher nest to foraging habitat ratio, which increases by fragmentation. This improves fitness and pollination coverage, but decreases pollination distance at the same time. Body size modulated this pattern with smaller bees benefitting more from fragmentation. In terms of traits, large bees have a disadvantage compared to small bees because they have to visit more flower for their pollen requirements (not compensated enough by velocity and short handling time) and wood-nesting bees have a disadvantage because they are limited where they can nest in the landscape and therefore need longer foraging distances. We found that landscape structure clearly affected bees and that improving the ratio of nest to foraging habitat by improving nest opportunities in large fields increases bee fitness and pollination services.

## Supporting information

S1 Appendix Appendix A: ODD protocol for the model SOLBEE (Overview, design concepts and details). (DOC)

S2 Appendix Appendix B: Supplementary figures (Effect of replicate simulations, responses as function of time investment per brood cell). (DOC)

S3 Appendix Appendix C: Adjusted vegetation (Simulation results). (DOC)

S1 Data Simulated data on which the analyses were performed. (ZIP)

S1 Model Model solbee full code: Includes a full verbal description of the model rules independent of programming language (pseudocode), the full model code in c++ and notes on how to compile it and test it. (ZIP)
