# Supplementary material for: Fragmentation of nest and foraging habitat affects time budgets of solitary bees, their fitness and pollination services, depending on traits: Results from an individual-based model
Source: PLoS One. 2018 Feb 14;13(2):e0188269. doi: 10.1371/journal.pone.0188269 (PMC5812554; doi:10.1371/journal.pone.0188269)
Supplement: S1 Appendix — (DOC) [file pone.0188269.s001.doc]

#### ODD protocol for the model SOLBEE (Overview, design concepts and details)

A more detailed version with more methodical considerations, data and references can be found in the book *In Silico Bees* (Everaars and Dormann, 2013) and in an earlier version of the protocol (Everaars, 2012).

**1. Purpose of the model**

This rule- and individual-based model simulates spatial pollen foraging behavior of solitary bees. Solitary bees are central-place foragers that forage for nectar and pollen without help of other individuals. Most of them nest either in the soil or in cavities in wood and they span a wide range of body sizes. Especially body size affects traits, performance and foraging behavior and these bees can be expected to respond differently to landscape structures. The aims of the model are: 1) understanding how wild bees with different traits interact with landscape structure by comparing the performance of groups of individuals 2) to give insight into the solitary bee's perspective of a landscape. The model is designed to be generic and to be applicable to multiple solitary bee species, which are defined by a wide range of parameters (traits required for pollen foraging).

**2. Entities, state variables and scales**

The model comprises a spatially explicit grid-based landscape with a given resource distribution and a community of pollen-collecting bees. Table 1 shows an overview of the parameters (*italic* in the text) and their possible values.

*The landscape (environment and spatial units)*

The model landscape is described in two steps with different resolution. We use a landscape generator (midpoint displacement algorithm`, Saupe, 1988) to stochastically (*landscape stochastic factor*) generate a course grid (grain size defined in *landscape element size*). This coarse grid has a given proportion of vegetation units suitable for foraging (*foraging habitat availability*) and the algorithm separates vegetation units from non-vegetation units with a given *landscape fragmentation*. We rescale the landscape to a finer resolution (grain size defined in *landscape detail*) to define small homogeneous habitat units that can be visited by foraging bees.

We chose our scales according to the ecology of solitary bees. We use a spatial extent of 1 km (*landscape size*) because solitary bees respond to landscape structures on scales up to 1000 m (Steffan-Dewenter et al., 2001). A coarse grain size of 50 m (*landscape element size*) results in “block-like” structures that mimic raster-based land-use maps in agriculturally-dominated landscapes and is a generally used grain size in landscape-scale studies with solitary bees (Monsevičius 1995; Williams & Tepedino 2003). The finest grain size (*landscape detail*) of 5 m results in a grid with 200 by 200 cells. Bees perceive landscape structures with nest substrates and foraging habitat in such fine detail (Artz & Waddington 2006; Franzén, Larsson & Nilsson 2009; Lonsdorf *et al.* 2009 ) and forage at a fine scales (e.g. Joshi *et al.* 2006; Diekötter *et al.* 2007).

Vegetated habitat units are described by *flower density*, average amount of *pollen per flower*, and the proportion of pollen that is available per pollinator visit (*pollen availability*, limits instant flower depletion). These fixed values describe the fine detail of each habitat grid cell that is needed at the individual bee level for pollen uptake and flying between flowers. The foraging habitat is further split up in edge and interior to have a simplified representation of the two different nesting preferences (wood nesting bees nest in woody substrates in field edges with shrubs and trees and soil-nesting bees nest in the soil at bare spots in the vegetation). We defined these field edges as 5 m wide strips (in line with *landscape detail*) and they are used for nesting and foraging by both wood-nesting and soil-nesting bees, while the interior is used for nesting by soil-nesting bees only and for foraging by both bee types. The *landscape quality* for bees is used to regulate initial bee densities in the landscape (section 5, initializations). Auxiliary parameters describe the average distance between flowers and the initial amount of pollen for each habitat unit. We track the remaining pollen volume and the number of bee visits per grid cell during simulation. The time horizon (*flytime*) is 14,400 time steps of one second (foraging period of four hours). This allows to model behavioral units that last from one to several seconds.

*The bees (the individuals)*

A bee is characterized by a certain size (*body length*) and a *nesting preference* based on substrate preference: above ground nesting (in wood and cavities) and subterranean nesting (in soil), two preferences hereafter referred to as wood-nesting and soil-nesting. Nesting preference determines the fixed nest location (central place foraging) for each individual bee. Empirical allometric rules (Table 3) are applied to link foraging traits (*pollen per brood cell*, *general return distance*, *far return distance*, *velocity medium/low*, *velocity high*, *handling time per flower*), and subsequently derived traits (*pollen capacity per bee*, *perception distance, length of flight units*) to body size. Other behavioral traits needed foraging (*flower memory, habitat cell memory, upper patch leaving threshold, lower patch leaving threshold, flight path tortuosity, ignorance, time at the nest*) were also parameterized with literature data (Everaars and Dormann, 2013). All traits remain constant during simulation.

Different state variables are recorded for each foraging trip for each bee: pollen collected during the trip, distance from the nest, current spatial location, number of unsuccessful and successful flower visits per trip, most recently visited locations, quality of last visited location, flight direction, start time of the foraging trip and "future time" to schedule the next behavioral element. At nest return, foraging trip data is collected (per bee): the number of returns to the nest, pollen delivered to the nest (volume per trip), number of flowers probed, maximum distance from the nest, and time spent per behavioral module. Other auxiliary variables keep track of the total distance flown and trip duration. At the end of the simulation the total pollen volume is converted into the (size related) number of brood cells (offspring proxy, no further population level variables) for each bee (Table 2, equation 3). These performance variables are output variables and averaged over all bees in the community.

Table 1/Table A. List of input variables and parameters used in the model with definitions and biological parameter ranges. Parameters with asterisk are expected to have an high impact or have a high level of uncertainty and they have been explored in a global perturbation analysis (Everaars and Dormann, 2013).

| Parameter | Unit | Definition | Biological range |
| --- | --- | --- | --- |
| Landscape: |  |  |  |
| landscape stochastic factor* |  | initial number for pseudo-random number generator |  |
| landscape size | km | spatial extent of the simulated landscape, length |  |
| landscape element size | m | length of most detailed landscape element (grain size of coarse grid) |  |
| landscape detail | m | grain size of the final grid |  |
| foraging habitat availability* |  | proportion of the landscape that is suitable foraging habitat |  |
| landscape fragmentation* |  | reverse of the Hurst exponent for "terrain smoothness", synonym with habitat fragmentation |  |
| flower density* | m^-2^ | number of flowers | 1 to 11000 |
| pollen per flower* | mm^3^ | pollen volume available in one flower during one day | 0.05 to 22.00 |
| pollen availability |  | proportion of pollen of a flower that is extractable per pollinator visit | <0.1 to 1.0, different per species, and relatively unknown |
| landscape quality* |  | maximum number of brood cells that can build from the available pollen in the landscape per individual | 10-100, real range unknown |
| Bee: |  |  |  |
| body length* | mm | body length of a bee | 4 to 26 |
| nesting preference* |  | category of nesting preference | wood nesting, soil nesting |
| flower memory* |  | min. number of most recently visited flowers that can be memorized | 1 to 20, real range unknown |
| habitat cell memory* |  | number of most recently visited habitat units that can be memorized | 3 to 50, real range unknown |
| flight path tortuosity* |  | density parameter of the wrapped Cauchy distribution that determines the relative amount of small turning angles during flight | 0.0 to 1.0, for bees unknown |
| lower patch leaving threshold |  | value of relative habitat cell quality below which a bee must leave the habitat grid cell | 0.0 to 1.0, real range unknown |
| upper patch leaving threshold |  | value of relative habitat cell quality above which a bee must stay in the habitat grid cell | 0.0 to 1.0, real range unknown |
| time at the nest* | s | time spent at the nest for non-foraging activities | 30 to 800 |
| flytime | s | total time of activity during a foraging day | 4 hours to 16 hours |
| Bee (dependent): |  |  |  |
| pollen per brood cell | mm^3^ | pollen volume that is needed to build one brood cell | scaling relationship |
| pollen capacity per bee | mm^3^ | max. amount of pollen that can be carried per foraging bout per bee | scaling relationship |
| velocity medium/low | m∙s^-1^ | flight velocity for flying in suitable habitat | scaling relationship |
| velocity high | m∙s^-1^ | flight velocity for flying in unsuitable habitat | scaling relationship |
| handling time per flower | s | time needed to remove pollen from flower | scaling relationship |
| perception distance* | m | distance radius within bees recognize habitat cells with flowers | scaling relationship |
| length of flight units* | m | mean length of a flight unit of which a flight path is built | scaling relationship |
| general return distance | km | the distance for which the probability of returning is 50% | scaling relationship |
| far return distance | km | the distance for which the probability of returning is 90% | scaling relationship |
| ignorance |  | probability of (non)ignoring a flower location within sight or at the present location, the inverse of habitat cell memory | 0.0 to 1.0, real range unknown |

Table 2/Table B. Overview of time penalties during the foraging cycle.

| Behavior | time penalties for: | value (minimal 1 s) |
| --- | --- | --- |
| 1. Forage flowers | poor habitat grid cell:  - assessing patch quality  good habitat grid cell:  - flying to a flower  - full flower: removing pollen from a flower  - empty flower: assessing flower | 1 s  based on medium/low velocity (size)  based on handling time (size)  1 s |
| 2. Neighboring cell | - accepting or denying a surrounding cell  - flying to a surrounding cell | 1 s  based on medium/low velocity (size) |
| 3. Fly around | - distance flown per flight unit | based on high velocity (size) |
| 4. Fly back | - distance flown per flight unit | based on high velocity (size) |
| 5. Nest reached | - pollen deposition and other non-foraging activities | 30 s (parameter *time at the nest*) |

Table 3/Table C. Allometric rules that were used in the model. All bee traits (y) are directly or indirectly related to body size (x) and in the model all are determined by the variable *body length*. Parameters are explained in Table 1.

|  | y | x | formula | data source |
| --- | --- | --- | --- | --- |
| 1 | dry body mass (mg) | body length (mm) | y = 0.0398∙x^2.589^ | (data taken from Müller et al., 2006) |
| 2 | intertegular-span (mm) | dry body mass (mg) | y = 0.77∙x^0.405^ | (Cane, 1987) |
| 3 | pollen per brood cell (mm^3^) | dry body mass (mg) | y = 10^(0.433+ 0.868∙ log_10_ x) | (Müller et al., 2006) |
| 4 | pollen capacity per bee (mm^3^) | pollen per brood cell (mm^3^) | y = x/10 | 10 trips, (Everaars and Dormann, 2013) |
| 5 | velocity medium/low (m∙s^-1^) | body length (mm) | y = −0.214+0.135∙x | (Everaars and Dormann, 2013) |
| 6 | velocity high (m∙s^-1^) | body length (mm) | y = 1.48+0.218∙x | (Everaars and Dormann, 2013) |
| 7 | handling time per flower (s) | body length (mm) | y = 919.62∙x^−1.914^ | (Everaars and Dormann, 2013) |
| 8 | perception distance (m) | velocity high (m∙s^-1^) | y = 10∙x | distance of ten seconds flight forward |
| 9 | perception area (m^2^) | perception distance (m) | y = (2∙x)^2^ | search window around the bee |
| 10 | length of flight units (m) | perception distance (m) | y = x/2 | perception distance flown in 2 flight units |
| 11 | general return distance (km) | intertegular-span (mm) | y = 10^(−1.643+3.242∙ log_10_ x) | (Greenleaf et al., 2007) |
| 12 | far return distance (km) | intertegular-span (mm) | y = 10^(−1.363+3.366∙ log_10_ x) | (Greenleaf et al., 2007) |

Table 4/Table D. Body size-based calculation of individuals and maximum flight distance on a foraging trip. Row labels (1-3) are used in the text.

|  | y | multiple x | formula | notes |
| --- | --- | --- | --- | --- |
| 1 | individuals | foraging habitat availability (am), landscape area, flower density (fd), pollen per flower (ppf), landscape quality (bdc), pollen per brood cell (ppb) | (*am* ∙ total area ∙ *fd* ∙ *ppf*) /  (bdc ∙ ppb) | Pollen volume available to the entire bee community (numerator) and the pollen volume available to one bee (denominator) are calculated in cm^3^. The total area is 1 km^2^. |
| 2 | probability  (of reaching a distance from the nest without knowledge of the environment, homing ability) | general return distance (r50), far return distance (r90),  distance from the nest | (distance−*shift*)/  (*Km*+distance−*shift*) | *shift*=1.125∙*r50*−0.125∙*r90*  Km=r50+shift |
| 3 | maximum distance from nest  (allowed to fly per foraging trip) |  | (U∙*Km*/1−U)+*shift* | Inverse of distance probability; U is drawn from a uniform distribution. |

**3. Process overview and scheduling**

The initialization phase consists of generating a landscape with foraging habitat and defining a bee community. The traits of each bee are based on their body size (section 5, allometric scaling), the nest location of each bee is assigned randomly according to rules and nesting preference. The number of bees is calculated based on several resource parameters (section 5, individuals). After initialization, a foraging day starts for each individual with five types of behavior in a foraging cycle (modified after Westphal et al., 2006). The behavior of one individual is strictly sequential in time, and lasts at least for one second (discrete time steps). When an individual performs a behavioral type, state variables recording foraging performance are changed. Individuals are processed in random order (list of bees randomized at each time step). For each behavior longer than one second the individual is excluded from processing for this time, which implies asynchronous updating of individuals. These behavioral modules are performed in a loop until a certain foraging time (*flytime*) is completed after which all activities are stopped instantaneously (fair comparison between bees) and values are averaged per bee and written to an output file. During the behaviors 1, 3 and 4 bees leave visitation marks in the landscape (on each entering event of a foraging habitat grid cell or stepwise fly over event of matrix grid cells). Visitation marks of foraging habitat can be used as a measure of pollination potential. Time penalties for each bee are given in Table 2.

The five behaviors in the foraging cycle are:

1. FORAGE FLOWERS (Forage flowers within a landscape grid cell):

- A bee flies from flower to flower directly (spatially implicit), encounters stochastically a full or empty flower (based on present resources), collects pollen, and decides about leaving the grid cell.
- Behavioral states can change into FLY AROUND (3) (current resources very low) and NEIGHBORING CELL (2) (current resources low) or FLY BACK (4) (enough pollen collected).

1. NEIGHBORING CELL (Fly to a suitable neighboring landscape grid cell)

- The bee considers the eight neighboring landscape grid cells for foraging. It accepts one of them randomly when it contains flowers and if it has not been visited recently.
- The behavioral state changes back to FORAGE FLOWERS (1) after moving there. On rejection of all eight cells, the behavioral state changes into FLY BACK (4) (far from the nest) or in FLY AROUND (3).

1. FLY AROUND (Fly around and look for unknown foraging areas)

- The bee performs a correlated random walk (changes direction and moves one step).
- The behavioral state changes to FORAGE FLOWERS (1) if either the new landscape grid cell is suitable (contains flowers and has not been visited recently) or if a suitable landscape grid cell has come within sight (move second step) or it can change into FLY BACK (4) (too far from the nest).
- With a certain probability suitable cells are ignored, leading to a more realistic foraging behavior (better matrix crossing and better foraging of interior habitat, see "pattern oriented modeling" section 8).

1. FLY BACK (Fly back to nest)

- The bee performs a directed random walk (correlated random walk in the direction of the nest).
- Eventually the state changes into NEST REACHED (5).

1. NEST REACHED

- The bee delivers the pollen to the nest and spends time on non-foraging activities (while other bees still deplete the landscape).
- The behavioral state is set to FORAGE FLOWERS (1) again.

**4. Design concepts**

*Basic principles*

We use several known foraging principles. We do not explore or compare the used principles; they only serve realistic foraging behavior. Bees use area restricted search to find resource items (e.g. Pyke, 1983) as a result of a limited knowledge and memory of the environment. They "scan" the local environment with a fixed-sized search window, inevitably encountering near resource patches first (Pyke, 1983). We also implemented "departure rules" or a patch "giving up time" (e.g. Charnov, 1976), based on local resource levels and thresholds, which is probably more realistic than time-based departure rules (Pyke, 1983). We used resource ratios to determine departure (Basset et al., 2002), based on actually probed flowers and the expected resource level. This enables individual bees to respond to changing resource levels caused by other foraging individuals. We introduced the concept of near-far foraging to get more realistic foraging behavior which is relevant in large patchy environments (Motro and Shmida, 1995), shown for solitary bees (Williams & Tepedino 2003; Beil, Horn & Schwabe 2008). Not considered concepts include "majoring and minoring" (Heinrich, 1979), the "Matching Law" and the "Ideal free distribution" (e.g. Thuijsman et al., 1995). They are known for nectar foraging honeybees or bumblebees but are not likely to apply to oligolectic pollen foraging solitary bees.

*Emergence*

The five different behavioral modes of the foraging cycle are combined in a sequence that depends on resource levels and location of the bee. Different individuals follow therefore different sequences (i.e. different behaviors coexist) and perform differently. The amount of pollen collected (or number of brood cells), the mean distance flown from the nest (or foraging distance) and the number of flowers visited are not coded into the model, but emerge from the sequence bees follow. Also a spatial "visitation map" emerges from behavioral rules and landscape structure and is used to calculate the percentage of the foraging habitat that has been visited by a bee community.

*Adaptation*

The individuals adapt to changing local resource conditions. After visiting and memorizing the status of a pre-defined minimal number of flowers (*flower memory*), the habitat grid cell quality (ratio of full to empty flowers encountered) is compared with the quality of the last visited habitat grid cell, acting as the actual resource expectation. According to the relative quality between both, and an upper and lower patch leaving threshold, a bee stays (relative quality of current grid cell better than upper patch leaving threshold) or leaves.

*Objectives*

We measure the performance of an individual by how much pollen it can collect within a fixed time span, constrained by time penalties for each behavioral rule (Table 2). Efficiency (and hence fitness) can for solitary bees be formulated as "potential number of offspring produced from the pollen per unit time on the flower" (Eickwort and Ginsberg, 1980), represented in the model by the number of brood cells (calculated from collected pollen and body size).

*Learning*

The behavioral rules are static without learning component (i.e. the same conditions in later foraging trips lead to the same decisions) because solitary bees are considered primitive foragers and display less efficient behavior than honeybees (Campan and Lehrer, 2002). The memory of the bees is used for memorizing locations and quality, and changes constantly (limited memory size), but the application of this memory is not optimized by learning. The sequence of behavioral modes and the outcome of decisions change with time as a result of local depletion of flowers and patches.

*Prediction*

The individuals cannot predict any future condition, except for the fact that they memorize recently visited habitat cells and avoid them because they predict that food condition are low there.

*Sensing*

Bees assess neighboring grid cells by vision and determine whether it has foraging resources and whether they have recently visited it. When flying around in unsuitable matrix, they visually assess the wider environment for foraging resources with a square-shaped search window (scaled with body size). Furthermore bees are aware of the distance from their nest and they can fly back anytime when they are too distant (calculated in the maximum distance allowed to fly per foraging trip, see section 7). The bees do not sense each other and experience local competition indirectly by declining resource levels.

*Interaction*

Interaction between individuals exists indirectly through competition for resources (continuously declining resource levels). The flowers around the nest are depleted first (but note the gradual release of pollen) and local competition pressure is influenced by the spatial allocation of nests in the landscape (determined by landscape structure and nesting preference). Therefore, the magnitude of interaction differs between landscapes, bee types and within a simulation in space and time.

*Stochasticity*

We used stochastic simulation in the following ways:

1. To generate landscapes: The fractal algorithm is partly based on stochastically generated noise.

2. To randomize the sequence and decisions of bees: We randomize the sequence of individuals at each time step, assign a random direction at start of foraging trip, choose randomly from suitable habitat cells around, and choose randomly when there are more "nearest locations" within sight.

3. To produce natural variability in behavior and nesting: Natural variability is a major element of the correlated random walk (wrapped Cauchy distribution for turning angles and a normal distribution for flight unit lengths) and different leaving thresholds. Nest sites are selected randomly. A landscape grid cell is drawn randomly until one is suitable for nesting according to different nesting preferences. Furthermore, we use specified frequencies (or a specified fraction) for ignoring suitable habitat grid cells, for determining the binary full or empty status of a flower, for clumping of nests (soil-nesting bees), and for the probability distribution of maximum return distances. In specific cases we use stochasticity for rounding to integers (when the frequency distribution between two is not uniform).

*Collectives*

We do not use collectives in our model. Each bee forages solitarily without direct interaction to other bees.

*Observation*

We record several performance measures for each individual bee: total pollen collected, realized (maximum) and mean foraging range (distance flown from the nest), number of returns to the nest and mean trip duration. For each behavioral module we recorded the amount of time spent in it. As output variables we use the arithmetic mean over all individuals and the standard deviation. At the landscape level we record total number of flower visits and the number of visitation marks per grid cell (section 3), which includes the binary state (visited, non-visited). From these visitation marks we can calculate the percentage that was visited (of all grid cells or of grid cells with foraging habitat). The mean number of flower visits per bee is a non-spatial but bee-centered pollination measure of how often pollen could be dislocated (but due to aggregation of full and empty flower visits not a performance measure of the bee).

**5. Initializations**

*Landscapes and habitat*

We use "noise" from a random number generator (*landscape stochastic factor*) and a Hurst exponent for fractional Brownian motion (*landscape fragmentation*) and a threshold (*foraging habitat availability*) to generate a landscape (Saupe, 1988). We generate landscapes that are symmetrically and wrapped (With et al., 1997). Symmetry prevents the entire foraging habitat to be in one corner, and wrapped boundaries give the landscape the appearance to be part of a larger landscape.

The model landscape has reflecting boundaries (rather than absorbing ones), because we deal with central-place foragers that have to return to their nests. To prevent increased local competition by the reflecting boundaries (effect low in large landscapes), we additionally prevented nesting at the border of the landscape. The boundary strip in which bees do not nest depends on body size (equals body length as being given in meters instead of mm, i.e. multiplied by 1000, rounded to full landscape grid cells), and slightly reduces the total area for nest habitat.

Suitable habitat cells are assigned an initial pollen volume based on *flower density* and *pollen per flower*. We calculate the proportion of available nest habitat (landscape grid cells suitable for nesting) according to the nesting preference of the bee. We do not assess other landscape metrics for the virtual landscapes, because most landscape metrics are highly correlated in artificial landscapes (Hargis et al., 1998).

*Allometric scaling*

Body size determines several foraging traits, described by allometric rules (Table 3). Conversion between body length, dry body mass (data taken from Müller et al., 2006) and intertegular-span (shortest linear distance between the wing tegulae, Cane, 1987) allows using different allometric relationships from the literature. Several traits are directly based on a certain publication (*pollen per brood cell*, *general return distance*, *far return distance*) while others were calculated with own collected data from multiple studies (*velocity medium/low*, *velocity high*, *handling time per flower*). We use these parameters subsequently to calculate *pollen capacity per bee*, *perception distance* (and perception area) and *length of flight units* (Table 3). We use allometric scaling of homing distance (*typical return distance* and *far return distance*) from the literature (Greenleaf et al., 2007) to calculate the maximum distance allowed to fly per foraging trip (see section 7 for details).

*Individuals*

We assume our bee community to be in balance with the available resources. In natural communities bee density is often related to flower cover (Banaszak, 2000; Calabuig, 2000) or flower diversity (Pawlikowski 1989; Gathmann, Greiler & Tscharntke 1994). We used pollen volume in the landscape as resource parameter (Müller et al., 2006) and scale the number of individuals to it. More specifically, the total number of individuals is calculated by dividing the pollen volume present in the landscape by the pollen volume available per individual (Table 4). The latter is calculated with the help of the parameter *landscape quality for bees* (or “bee density control”) as the "potential offspring (brood cells) per individual", which is independent of body size. Hence, the number of individuals is dependent on size, since small bees require less pollen to build the same number of brood cells. This offspring perspective on the individual level ensures equal performance potential for all bees, which makes all bee types comparable. The chosen value (30) for *landscape quality for bees* results in realistic bee densities in the landscape (Everaars and Dormann, 2013) with high bee numbers for small bees and lower numbers for large bees and increases with increasing foraging habitat in the landscape. A disadvantage of this approach is that model runtime varies several orders of magnitude for different parameter settings (i.e. *body length* and *foraging habitat availability* as well as *flower density* and *pollen per flower*). The initially calculated number of individuals (in current model version on basis of a bee community of bees of the same size) remains constant during simulation (one foraging day without population dynamics).

Each individual is initialized with a nest location, a random direction (used in the behavior *fly around*) and a maximum distance allowed to fly (see section 7 details). Each individual is assigned a nest location near foraging resources (Westrich, 1996). Wood-nesting bees accept a (randomly chosen) location to nest when it is at the edge of the foraging habitat and soil-nesting bees accept a location all over the foraging habitat. Soil-nesting bees preferably nest in the vicinity of other bee nests, since the best substrates occur clumped and aggregated nesting improves mate finding (Eickwort & Ginsberg 1980; Cane 1991; Neff & Danforth 1991; Julier & Roulston 2009). Hence, we implemented moderate nest clumping for soil-nesting bees to generate a more realistic nest distribution (not further analyzed, we investigated difference of two nesting preferences *per se*). Random habitat grid cells are accepted for nesting when there is already a bee nest, or when a neighboring grid cell has a nest (with 90% probability), or when a neighboring cell has no nest (with 10% probability).

**6. Input data**

The model does not use input data to represent time-varying processes.

**7. Details**

This section describes several implementation details of concepts introduced in the preceding paragraphs.

*Patch leaving rules and flower encountering*

The probability of leaving a grid cell with foraging habitat is based on the relative quality of the habitat grid cell compared with a previously visited habitat grid cell. Habitat grid cell quality is defined as the fraction of successful flower visits (fraction full flowers of all flower visits since entering the grid cell, information that is valid after visiting a minimal number of flowers, *flower memory*). The relative quality is then calculated by dividing current habitat quality by the remembered habitat quality. If the current grid cell has at least the same quality as the previous visited habitat grid cell (value of 1.0 or higher) and the *upper patch leaving threshold* is set to 1.0, the probability of leaving is 0.0. If the relative cell quality comes below the *lower patch leaving threshold* (a value of 0.5 means threshold at half of the quality of the previous grid cell), the probability of leaving is 1.0 and the bee flies up in search for a better location (behavior FLY AROUND). When the relative habitat quality is between the upper and lower patch leaving threshold, a third leaving threshold is calculated (uniformly distributed between both thresholds, determined stochastically). If the relative habitat grid cell quality is below this third threshold, the bee will search for resources in neighboring grid cells (behavior NEIGBORING CELL); above this threshold it will stay.

We did not track the pollen volume of each flower it the landscape, but the amount of pollen remaining per grid cell, as approximation for the amount of full flowers. We divide the remaining pollen over the pollen volume per flower and then over the number of flowers per grid cell (defined by *flower density*) to estimate the proportion of full flowers in that grid cell. The estimated proportion of full flowers is used to determine stochastically (by probability) whether a flower visit was successful (full) or not. If a full flower is encountered, the bee takes up pollen according to the pollen volume available from a full flower (defined by *pollen per flower* and *pollen availability*) and the remaining capacity of the bee (based on previous flower visits and *pollen capacity per bee* related to *body length*). The amount of pollen in that grid cell decreases by the amount taken up by the bee. The bee receives a time penalty for flying to the flower (based on distance and *medium velocity*, for both full and empty flowers) and for collecting the pollen (*handling time*).

*Maximum distance allowed to fly on a foraging trip (distance of certain return)*

We use allometric scaling of homing distance, or preferred return distance (*typical return distance* and *far return distance*), from the literature (Greenleaf et al., 2007) to calculate the maximum distance allowed to fly per foraging trip. The typical homing distance is defined as the distance where 50% of the bees does not return home upon release at this distance, which means that 50% of the bees does not know this location. Homing distance does not only differ between species of different size, but also between individuals. Since maximum homing distance can be considered as the maximum distance with knowledge of the environment, it can be different for individuals and different for multiple flight directions from the nest. There is also a minimal knowledge of the environment (minimal distance from the nest for which a bee would always be able to find the nest back). Within this minimal distance from the nest, bees only return with a full pollen load. Beyond this minimal distance bees have no knowledge of the environment anymore, with a certain probability, and therefore return to the nest (homing) with an incomplete load. In case of a full pollen load closer to the nest these far distances are never reached. We calculate this maximum distance allowed to fly on a foraging trip in several steps.

- First we calculated the typical (r50) and far (r90) homing distance (distance beyond which 50% and 90% of the bees were not able to return at release respectively) for a bee of a certain size (table 3) based on a known allometric relationship (Greenleaf et al., 2007).
- In the next step, we calculate a full range of homing distances for a bee of a certain size based on this typical (r50) and far (r90) homing distance. We use a hyperbolic Michaelis–Menten function to describe a (saturating) homing probability curve based on two values (Table 4). We altered the Michaelis–Menten function by introducing a *shift* parameter (table 4, row 2) which represents a certain minimal knowledge of the environment (distance for which a bee would always be able to find the nest back). When 50% and 90% homing probability (belonging to typical and far homing distance, measured from the nest) share the same *shift* value, one gets the equation for *shift* (Table 4, row 2). This alters the saturation constant *Km* accordingly (Table 4, row 2). The return or homing probability can then be calculated as a function of distance from the nest (Table 4, row 2).
- We inversed this function (Table 4, row 3), giving a stochastically determined distance per foraging trip beyond a bee will not forage anymore and return (the distance at which beyond it has no knowledge of the environment in this direction). This reproduces a realistic set of distances from the nest where the bee should maximally return, never close to the nest and with a similar shape as for real foraging data (Abrol and Kapil, 1994). An asymmetrical S-Curve would probably be a good alternative, but we could not fit it with two known points.

*Introduction of parameter “ignorance’*

The recorded visitation pattern (grid cells where bees left one or more visitation marks) showed during development that an additional foraging parameter was required. Bees with area restricted search and a limited memory easily fly back to old patches (grid cells) in the model and almost never cross the matrix to reach more distant patches. We considered this behavior "too locally optimized" and added a parameter (*ignorance*) to define that a bee ignores decisions with a certain probability. This probability assumes an innate preference of solitary bees to occasionally take the risk of crossing the matrix for finding far resource patches with higher resource abundance instead of optimizing the short-term profit (near-far foraging, Williams & Tepedino 2003; Beil, Horn & Schwabe 2008). We related *ignorance* reciprocally to memory capacity (smaller memory induces more risky decisions, i.e. a *habitat cell memory* of 5 grid cells gives an *ignorance* of 0.2). The probability of ignorance (in our case 0.1) is applied in two cases in the behavior FLY AROUND: when a suitable grid cell is encountered (non-remembered grid cell with foraging habitat) and when an unsuitable grid cell is encountered (remembered grid cell or matrix grid cell) but a suitable grid cell is “in sight”. In these cases, the bee keeps flying further around. This addition results in a more plausible foraging pattern where bees sufficiently cross the matrix and search for better patches outside the local patch. Bees also visit more often the interior of flower field.

**References**

Abrol, D.P., Kapil, R.P., 1994. On homing ability and pollination effectiveness of bees. Mysore Journal of Agricultural Sciences 28, 249-252.

Banaszak, J., 2000. Effect of habitat heterogeneity on the diversity and density of pollinating insects, in: Ekbom, B.S., Irwin, M.E., Robert, Y. (eds.), Interchanges of Insects between Agricultural and Surrounding Landscapes. Kluwer Academic, Dordrecht, The Netherlands, pp. 123-140.

Basset, A., Fedele, M., DeAngelis, D.L., 2002. Optimal exploitation of spatially distributed trophic resources and population stability. Ecol. Model. 151, 245-260.

Calabuig, I., 2000. PhD thesis: Solitary bees and bumblebees in a Danish agricultural landscape, Department of Population Ecology. University of Copenhagen, Copenhagen, p. 103.

Campan, R., Lehrer, M., 2002. Discrimination of closed shapes by two species of bee, *Apis mellifera* and *Megachile rotundata*. J. Exp. Biol. 205, 559-572.

Cane, J.H., 1987. Estimation of bee size using intertegular span (Apoidea). J. Kans. Entomol. Soc. 60, 145-147.

Charnov, E.L., 1976. Optimal foraging, marginal value theorem. Theor. Popul. Biol. 9, 129-136.

Eickwort, G.C., Ginsberg, H.S., 1980. Foraging and mating-behavior in Apoidea. Annu. Rev. Entomol. 25, 421-446.

Everaars, J., 2012. The response of solitary bees to landscape configuration with focus on body size and nest-site preference. PhD Dissertation. Department of Zoology, Martin-Luther-Universität Halle-Wittenberg, p. 146.

Everaars, J., Dormann, C.F., 2013. A simulation model for non-*Apis* bees; solitary bees foraging and competing for pollen., in: Devillers, J. (ed.), In Silico Bees. CRC Press, pp. 209-268.

Greenleaf, S.S., Williams, N.M., Winfree, R., Kremen, C., 2007. Bee foraging ranges and their relationship to body size. Oecologia 153, 589-596.

Hargis, C.D., Bissonette, J.A., David, J.L., 1998. The behavior of landscape metrics commonly used in the study of habitat fragmentation. Landscape Ecol. 13, 167-186.

Heinrich, B., 1979. Majoring and minoring by foraging bumblebees, *Bombus vagans* - experimental analysis. Ecology 60, 245-255.

Motro, U., Shmida, A., 1995. Near-far search - an evolutionarily stable foraging strategy. J. Theor. Biol. 173, 15-22.

Müller, A., Diener, S., Schnyder, S., Stutz, K., Sedivy, C., Dorn, S., 2006. Quantitative pollen requirements of solitary bees: Implications for bee conservation and the evolution of bee-flower relationships. Biol. Conserv. 130, 604-615.

Pyke, G.H., 1983. Animal movements: An optimal foraging approach., in: Swingland, J.R., Greenwood, P.J. (eds.), The Ecology of Animal Movement. Oxford University Press, Oxford, pp. 7-31.

Saupe, D., 1988. Algorithms for random fractals, in: Peitgen, H.-O., Saupe, D. (eds.), The sciences of fractal images. Springer-Verlag, New York, pp. 71-113.

Steffan-Dewenter, I., Münzenberg, U., Tscharntke, T., 2001. Pollination, seed set and seed predation on a landscape scale. Proc. R. Soc. Lond., Ser. B: Biol. Sci. 268, 1685-1690.

Thuijsman, F., Peleg, B., Amitai, M., Shmida, A., 1995. Automata, matching and foraging behavior of bees. J. Theor. Biol. 175, 305-316.

Westphal, C., Steffan-Dewenter, I., Tscharntke, T., 2006. Foraging trip duration of bumblebees in relation to landscape-wide resource availability. Ecol. Entomol. 31, 389-394.

Westrich, P., 1996. Habitat requirements of central European bees and the problems of partial habitats., in: Matheson, A., Buchmann, S.L., O'Toole, C., Westrich, P., Williams, I.H. (eds.), The Conservation of Bees. 18 ed. Academic Press, London, pp. 1-16.

With, K.A., Gardner, R.H., Turner, M.G., 1997. Landscape connectivity and population distributions in heterogeneous environments. Oikos 78, 151-169.
