# Supplementary material for: Fragmentation of nest and foraging habitat affects time budgets of solitary bees, their fitness and pollination services, depending on traits: Results from an individual-based model
Source: PLoS One. 2018 Feb 14;13(2):e0188269. doi: 10.1371/journal.pone.0188269 (PMC5812554; doi:10.1371/journal.pone.0188269)
Supplement: S2 Appendix — (DOC) [file pone.0188269.s002.doc]

Supplementary figures


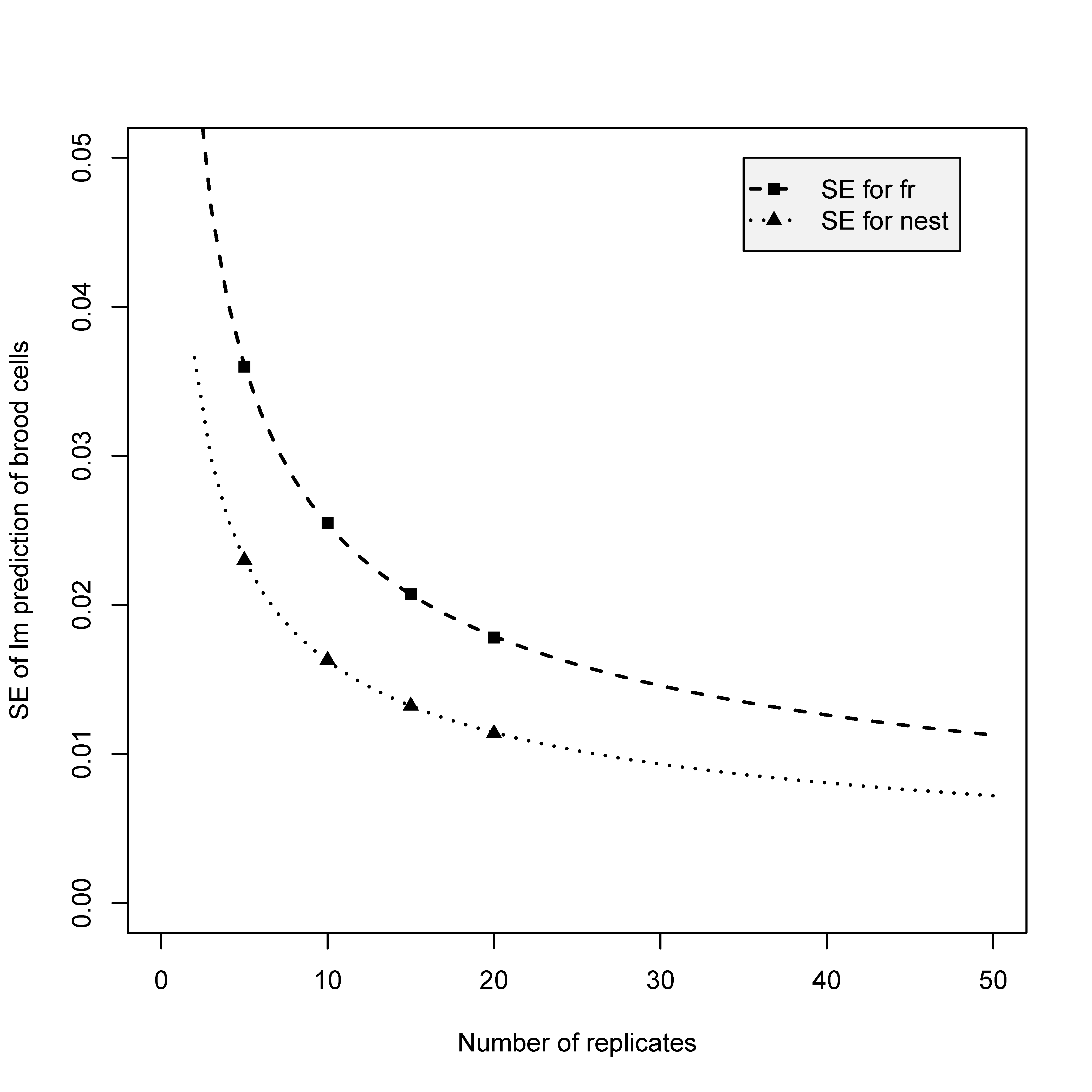


Figure A. Effect of replicate simulations, a general pattern exemplified by selected response and predictor. Standard error of two selected model predictors (*landscape fragmentation* and *nesting preference* from linear regression models predicting the simulated number of brood cells, based on subsequently more stochastic replicates (steps of 5).


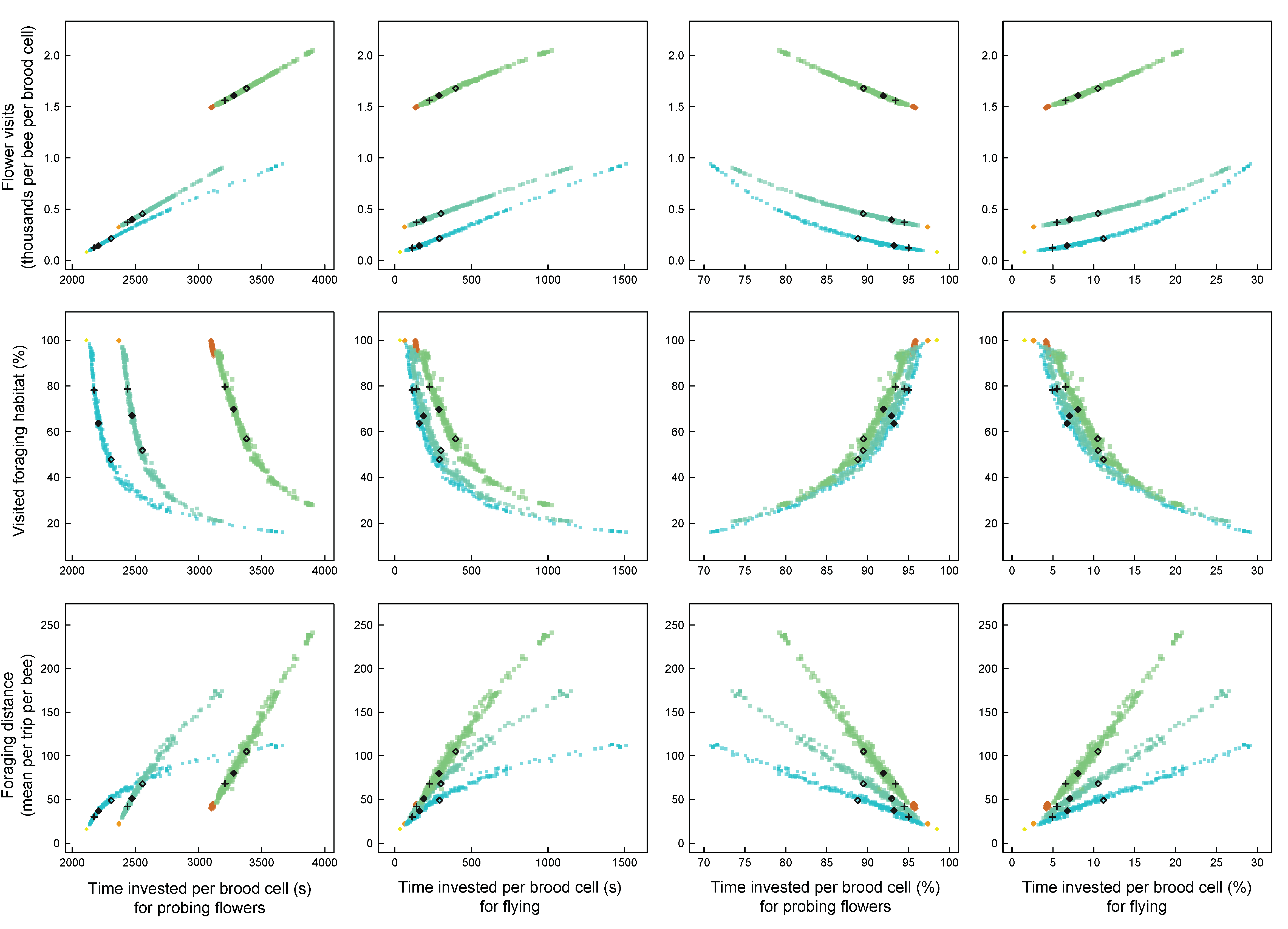


Figure B. Time investment per brood cell in seconds and in percentage of total time invested per brood cell, for the number of flower visits per brood cell, visited foraging habitat and mean foraging distance. Time investment is given for probing flowers (time spent in FORAGE FLOWERS per brood cell) and for flying (combined time spent in FLY AROUND, FLY BACK and NEIGHBORING CELL, per brood cell).
