# Supplementary material for: Fragmentation of nest and foraging habitat affects time budgets of solitary bees, their fitness and pollination services, depending on traits: Results from an individual-based model
Source: PLoS One. 2018 Feb 14;13(2):e0188269. doi: 10.1371/journal.pone.0188269 (PMC5812554; doi:10.1371/journal.pone.0188269)
Supplement: S3 Appendix — (DOC) [file pone.0188269.s003.doc]

Adjusted vegetation

Table A. Analysis of variance for the number of brood cells, flower visits, visited foraging habitat and foraging distance in response to landscape fragmentation based on 20 replicates (12,000 simulations). Further predictors were landscape type (foraging habitat availability), the bees' body length and nesting preference and all interactions. Given are F-value, % explained, the used degrees of freedom (used df), remaining degrees of freedom (residual df) and adjusted R^2^. The × indicates an interaction between two parameters.

|  | Response | | | | | | | |
| --- | --- | --- | --- | --- | --- | --- | --- | --- |
|  | Brood cells | | Flower visits | | Visited foraging habitat | | Foraging distance | |
| Predictors | % Explained | F value | % Explained | F value | % Explained | F value | % Explained | F value |
| landscape fragmentation | 0.9 | 461.9 | 0.0 | 120.8 | 1.5 | 840.4 | 0.9 | 356.2 |
| foraging habitat availability | 9.3 | 4584.1 | 0.4 | 1818.7 | 18.3 | 9962.9 | 13.8 | 5683.9 |
| body size | 29.7 | 7311.5 | 95.6 | 223364.7 | 0.7 | 189.6 | 21.4 | 4410.2 |
| nesting preference | 38.5 | 18986.4 | 1.2 | 5709.8 | 52.9 | 28845.4 | 40.8 | 16797.9 |
| landscape fragmentation × foraging habitat availability | 0.1 | 24.7 | 0.0 | 10.0 | 0.1 | 45.8 | 0.0 | 17.5 |
| landscape fragmentation × body size | 0.1 | 23.2 | 0.0 | 69.3 | 0.0 | 4.1 | 0.0 | 9.2 |
| landscape fragmentation × nesting preference | 1.0 | 473.1 | 0.0 | 121.4 | 1.6 | 851.3 | 0.8 | 332.5 |
| foraging habitat availability × body size | 1.1 | 264.7 | 0.4 | 820.6 | 0.2 | 66.2 | 1.1 | 225.9 |
| foraging habitat availability × nesting preference | 9.4 | 4629.4 | 0.4 | 1787.0 | 18.3 | 9951.4 | 12.3 | 5079.4 |
| body size × nesting preference | 3.9 | 964.5 | 1.3 | 3078.4 | 1.0 | 259.6 | 1.5 | 315.9 |
| Residuals | 6.1 |  | 0.6 |  | 5.5 |  | 7.2 |  |
| *df used / residual df* | *14* | *2985* | *14* | *2985* | *14* | *2985* | *14* | *2985* |
| *(adjusted) R ^2^* | *0.94* |  | *0.99* |  | *0.95* |  | *0.93* |  |

Table B. Analysis of variance for the number of brood cells, flower visits, visited foraging habitat and foraging distance (top to bottom) in response to one of three focal predictors; nest habitat availability, local bee density and ratio of nest to foraging habitat (left to right) based on 20 replicates. The layout of these twelve linear models follows the layout of the plots in figure 3. Body length is included as co-predictor, as well as quadratic terms (and cubic for visited foraging habitat) and their interactions (with × indicated). Given are F-value and % explained. The used degrees of freedom (used df), remaining degrees of freedom (residual df) and adjusted R^2^ are given additionally for each model in *italic*.

|  |  | Focal predictor | | | | | |
| --- | --- | --- | --- | --- | --- | --- | --- |
|  |  | 1. Nest habitat availability (log) | | 2. Local bee density (log) | | 3. Ratio of nest to foraging habitat (log) | |
| Response | Predictors | % Explained | F value | % Explained | F value | % Explained | F value |
| Brood cells | Body size | 29.7 | 1619.8 | 29.7 | 73642.0 | 29.7 | 72830.0 |
|  | Focal predictor | 36.2 | 3954.4 | 66.2 | 328339.4 | 59.0 | 289407.6 |
|  | Focal predictor × body size | 2.5 | 274.0 | 1.8 | 8766.3 | 3.7 | 18375.3 |
|  | Focal predictor ^2^ | 3.9 | 212.1 | 1.0 | 2507.1 | 6.4 | 15806.5 |
|  | Focal predictor ^2^ × body size | 0.3 | 16.4 | 0.8 | 1940.5 | 0.6 | 1378.5 |
|  | Residuals | 27.4 |  | 0.6 |  | 0.6 |  |
|  | *df used / residual df* | 8 | 2991 | 8 | 2991 | 8 | 2991 |
|  | *(adjusted) R ^2^* | 0.73 |  | 0.99 |  | 0.99 |  |
| Flower visits | Body size | 95.6 | 75292.2 | 95.6 | 2842788.0 | 95.6 | 2824006.0 |
|  | Focal predictor | 1.1 | 1767.5 | 3.0 | 181173.4 | 2.0 | 116282.6 |
|  | Focal predictor × body size | 0.0 | 69.4 | 1.0 | 57851.6 | 0.1 | 7592.0 |
|  | Focal predictor ^2^ | 1.2 | 975.1 | 0.3 | 8500.5 | 2.1 | 61457.5 |
|  | Focal predictor ^2^ × body size | 0.1 | 64.2 | 0.0 | 876.6 | 0.2 | 4633.3 |
|  | Residuals | 1.9 |  | 0.1 |  | 0.1 |  |
|  | *df used / residual df* | 8 | 2991 | 8 | 2991 | 8 | 2991 |
|  | *(adjusted) R ^2^* | 0.98 |  | 1.00 |  | 1.00 |  |
| Visited foraging habitat | Body size | 0.7 | 21.9 | 0.7 | 438.8 | 0.7 | 464.7 |
|  | Focal predictor | 47.1 | 2965.9 | 88.8 | 112022.5 | 86.0 | 114969.9 |
|  | Focal predictor × body size | 1.7 | 108.0 | 0.0 | 0.8 | 6.5 | 8644.7 |
|  | Focal predictor ^2^ | 0.9 | 28.1 | 3.5 | 2201.2 | 1.8 | 1224.0 |
|  | Focal predictor ^2^ × body size | 0.0 | 0.5 | 3.3 | 2101.5 | 0.0 | 0.0 |
|  | Focal predictor ^3^ | 2.0 | 125.1 | 1.3 | 1681.3 | 2.6 | 3473.5 |
|  | Focal predictor ^3^ × body size | 0.1 | 4.4 | 0.0 | 5.4 | 0.1 | 89.5 |
|  | Residuals | 47.5 |  | 2.4 |  | 2.2 |  |
|  | *df used / residual df* | 11 | 2988 | 11 | 2988 | 11 | 2988 |
|  | *(adjusted) R ^2^* | 0.53 |  | 0.98 |  | 0.98 |  |
| Foraging distance | Body size | 21.4 | 915.7 | 21.4 | 70161.6 | 21.4 | 56954.0 |
|  | Focal predictor | 38.0 | 3247.1 | 58.2 | 381499.7 | 66.6 | 354213.6 |
|  | Focal predictor × body size | 4.1 | 348.9 | 3.9 | 25310.7 | 8.0 | 42320.7 |
|  | Focal predictor ^2^ | 1.3 | 54.1 | 15.6 | 51180.2 | 2.7 | 7251.9 |
|  | Focal predictor ^2^ × body size | 0.3 | 11.4 | 0.4 | 1281.9 | 0.7 | 1900.7 |
|  | Residuals | 35 |  | 0.5 |  | 0.6 |  |
|  | *df used / residual df* | 8 | 2991 | 8 | 2991 | 8 | 2991 |
|  | *(adjusted) R ^2^* | 0.65 |  | 1.00 |  | 0.99 |  |
