# Supplementary material for: Fragmentation of nest and foraging habitat affects time budgets of solitary bees, their fitness and pollination services, depending on traits: Results from an individual-based model
Source: PLoS One. 2018 Feb 14;13(2):e0188269. doi: 10.1371/journal.pone.0188269 (PMC5812554; doi:10.1371/journal.pone.0188269)
Supplement: S1 Model — (ZIP) [file pone.0188269.s005.zip › S5_Solbee_Full_Code/Full model rules.doc]

## Full rule description of the model SOLBEE and scheduling (including all submodels)

This contains the full verbal description of the model rules independent of programming language. We implemented these rules in C++, but they could be implemented in any programming language. We provide here also additional details that are required to reproduce the model but are not given elsewhere. We left out some initializations, resets (back to zero), safety checks, and observer parameters to keep the structure clear. They can be reproduced by common sense of the programmer (and are hence part of good programming practice rather than part of our rule set design). In two cases a value was technically not allowed to become zero (division by zero) so that a correction was needed and they are included in the following description. Steps marked with an * are explained in more detail separately, after 'Main'.

**A. Main**

Read input value from text file

Calculate secondary input values *

Generate landscape *

Define a list of bees of one species

Assign a nest to each bee *

I. Start time loop (for each time step do:)

Shuffle bee list randomly

II. Start individual loop

A. When an individual's future time equals current time step (do:)

(Initializations)

Action according to one of following 5 behavioural states (1. FORAGE FLOWERS, 2. NEIGHBOURING CELL, 3. FLY AROUND, 4. FLY BACK, 5. NEST REACHED):

1. FORAGE FLOWERS (Forage flowers within a landscape grid cell)

a. Perform only when newly entering the landscape cell:

Set visitation mark in the landscape;

Save position in memory (overwrite first position when memory is full).

b. Perform always:

aa. If minimal flowers to probe within landscape grid cell have been probed:

Calculate leave threshold (third threshold value, uniform between upper and lower threshold);

Calculate good fraction (success visits/flowers probed);

Compare this fraction with remembered fraction (thus ratio present fraction/remembered fraction, is >1 if better than remembered);

aaa. If ratio present-remembered < leave threshold:

Leave (aaaa or bbbb);

Set time to spend till next behaviour (1 s for assessing patch quality);

Add successful and unsuccessful visits from cell level to foraging bout level;

aaaa. If ratio present-remembered < lower threshold:

Set an initial direction to fly;

Leave; set behavioural state to FLY AROUND (3).

bbbb. If ratio present-remembered between lower threshold and upper threshold:

Leave; set behavioural state to NEIGHBOURING CELL (2).

bbb. If ratio present-remembered > leave threshold:

Stay, and continue with bb.

bb. (1) If minimal # flowers has not been probed yet or (2) if it proved to be a good cell (and individual stays, coming from aa.):

Visit a new flower:

Calculate time to spend till next behaviour (low velocity × average flower distance);

Calculate local floral resources (implicitly) *;

aaa. When a successful flower visit has been made:

add 1 to successful visits and total visits at cell level;

add pollen to individual (foraging bout level);

Determine time to spend till next behaviour (add handling time);

aaaa. If loading capacity is reached:

Leave:

Add pollen to total collected pollen (equals loading capacity, at foraging day level);

(Determine current distance from nest and realized foraging range);

Add successful and unsuccessful visits from cell level to bout level;

Leave; set behavioural state to FLY BACK (4).

bbbb. Loading capacity not reached:

Stay.

bbb. If a unsuccessful flower visit has been made:

Add 1 to unsuccessful visits and total visits at cell level;

Set time to spend till next behaviour (add minimal handling time).

2. NEIGHBOURING CELL (Fly to a suitable neighbouring landscape grid cell)

Analyze 8 neighbouring landscape grid cells *;

Determine time to spend till next behaviour (add 1 second for decision);

a. If a suitable cell is found:

Move and start foraging flowers there; set behavioural state to FORAGE FLOWERS (1);

Calculate time to spend till next behaviour (add high velocity × cell size).

b. If no suitable cell could be found:

Leave:

Determine distance from nest;

aa. If the maximum foraging range is reached:

(Determine realized foraging range);

Set maximum foraging distance for next foraging bout *;

Leave; set behavioural state to FLY BACK (4).

bb. If the maximum foraging range is not reached:

Set an initial direction to fly;

Leave; set behavioural state to FLY AROUND (3).

3. FLY AROUND (Fly around and look for unknown foraging areas)

Perform a "correlated random walk" (change direction and move one step with certain length, and correct for landscape border*);

Set visitation mark in the landscape;

Calculate time to spend till next behaviour (high velocity × distance moved);

a. If new landscape grid cell is foraging habitat and not remembered:

aa. With a high probability (1-"ignorance"):

Set behavioural state to FORAGE FLOWERS (1).

bb. With a low probability ("ignorance"):

Determine current distance from nest;

aaa. If the maximum foraging range is reached:

(Determine realized foraging range);

Set maximum foraging distance next foraging bout *;

(set pollen collected to non-zero (i.e. very low) if no pollen were collected);

Leave; set behavioural state to FLY BACK (4).

bbb. If the maximum foraging range is not reached:

Keep flying around next time.

b. If new cell is remembered (recently visited) or non-foraging habitat:

Determine whether there is a grid cell with foraging habitat within sight and if so choose closest *;

aa. If there is a landscape grid cell with foraging habitat in sight and this is not ignored (a high probability: 1-"ignorance"):

Fly to cell with foraging habitat (perform second move);

Calculate time to spend till next behaviour (add high velocity × distance moved);

Set behavioural state to FORAGE FLOWERS (1).

bb. If there is no landscape grid cell with foraging habitat in sight or foraging habitat is ignored (a low probability: "ignorance"):

Keep flying around next time.

4. FLY BACK (Fly back to nest)

Perform a "directed random walk" (move one step with certain length in the direction of the nest);

Set a visitation mark in the landscape;

Calculate time to spend till next behaviour (high velocity × distance moved);

a. If nest reached within this step:

Set behavioural state to NEST REACHED (5).

b. If nest not reached within this step:

Keep flying to nest next time.

5. NEST REACHED

Add flowers visited and pollen collected from bout level to global level;

Set time to spend till next behaviour (fixed time at nest);

Determine foraging bout duration and set new time for bout start;

Remember cell quality of last visited flower habitat cell (success visits/flowers probed) (set quality to non-zero (i.e. very low) in case it applies);

Do several resets (including memory of visited cells);

Add one to nest returns;

Set behavioural state to FORAGE FLOWERS (1).

After one of the behaviours:

Set wait time (time to spend till next behaviour, minimal 1 second which is the smallest time step);

Set future time step when to do something (current time step + wait time).

B. If an individual's future time is higher than current time step

Proceed with next individual.

All individuals are checked (end of loop II)

Foraging day completed (end of loop I)

Collecting data at the end of the foraging day

Individual loop 1: collate data per individual

a. If individual is not currently at nest:

Number of foraging bouts = nest visits+1;

Count additional flowers visited and pollen collected.

b. If individual is currently at nest:

Number of foraging bouts = nest visits.

Calculate average return distance and average trip duration;

Add 1 to non-returned individuals if individual never returned;

Add 1 to deaths if individual died;

Individual loop 2: average across individuals

Calculate totals and averages for all individuals;

Convert pollen collected into brood cells;

Calculate other describing statistics (e.g. mortality);

Collect landscape level data

(Calculate additional landscape and nest site statistics);

Write output files

Write all essential values to file (input values, describing landscape statistics, bee performance);

Write landscape map to two files (as graphical and textual format);

Write visitation map to two files (as graphical and textual format).

**B. Submodels (above marked with *)**

Calculate secondary input values (e.g. allometric scaling rules)

Set coarse grid dimensions and fine grid dimensions according to landscape extent and landscape element size or perceived detail by the bee;

Set size related bee characteristics (from body length and calculated weight and inter-tegular span):

pollen needed for one brood cell, maximum pollen load per foraging bout, high velocity, low velocity, flower handling time, sight distance, average flight 'step' length, border effects correction, number of individuals, curve characteristics for determining foraging range, pollen uptake per visit.

Generate landscape

Create three grids:

- a 'generator' grid with 257 × 257 cells for the landscape generator;

- a coarse grid, based on fixed landscape element detail;

- a fine grid, based on the bees perspective of the landscape;

Generate a landscape on the 'generator' grid with floating point numbers according to Saupe's midpoint displacing algorithm (with wrapping and symmetry) using the fragmentation value;

Resize (in our case up-sampling to 40 by 40 cells) this map to a coarse grid of floating point numbers

"flood the valleys" (after sorting cells) using a threshold value for foraging habitat availability (convert to binary suitable and unsuitable foraging habitat);

Resize the coarse grid to a fine grid for foraging bees (in our case down-sampling to 200 by 200 cells);

Initialize each foraging habitat cell with pollen according to flower density and pollen per flower.

Assign a nest to each bee

Choose a random location and correct for landscape border (no nesting);

a. If the bees nest in woody edge habitat:

Accept location if the chosen landscape grid cell is foraging habitat and has at least one neighbouring cell unsuitable for foraging.

b. If bees nest in bare soil habitat:

Accept location if the chosen landscape grid cell is foraging habitat and if:

- grid cell is already assigned as nesting habitat (at least one individual nesting in it);

- grid cell has a neighbouring cell assigned as nesting habitat and a certain probability applies (nest clumping);

- grid cell has no neighbouring cell assigned as nesting habitat and a certain probability applies (1-nest clumping).

If location not accepted, choose a new location, until one accepted (safety break for 1 million tries).

Calculate local floral resources

Determine remaining pollen in landscape grid cell (current floral resources);

Calculate the possible number of full flowers remaining (from pollen and flower density);

Calculate the proportion of full flowers (possible number of full flowers/initial number of flowers) currently in landscape grid cell (this is the probability of visiting a flower with pollen);

Determine success or failure;

If success, deplete pollen in landscape grid cell (calculate future floral resources) according to the pollen a bee gets (as determined in function 'calculate secondary input values' *).

Analyze 8 neighbouring landscape grid cells

Consider 3 by 3 landscape grid cells around current position;

Suitable if:

- foraging habitat, AND

- not current position, AND

- not outside landscape borders, AND

- not in memory.

Choose one of the suitable cells randomly, or return "unsuitable".

Set maximum foraging distance next foraging bout

Inverse function of the Michaelis-Menten Curve, with parameters as determined in function 'calculate secondary input values';

A uniform probability (input) determines the maximum foraging distance from the nest for the next bout.

Choose closest landscape grid cell with foraging habitat

Consider the area within the sight radius (square around current position);

Correct for landscape border * (rectangular);

Analyse landscape within this area (defined by 4 outside positions);

Determine closest cells with foraging habitat that is not memorized;

Choose one of the closest cells randomly.

Correct for landscape border (when flying)

If a flight step is supposed to go over the landscape border, take the same step in opposite direction (mirror move in x or y direction);

Calculate new direction (take position outside grid as 'old position' and take corrected position as 'new location').
